# Can formal home and community-based care substitute informal care? Evidence from Chinese Longitudinal Healthy Longevity Survey

**DOI:** 10.1186/s12877-024-05312-7

**Published:** 2024-09-03

**Authors:** Yixiao Wang, Bei Wu, Wei Yang

**Affiliations:** 1 School of Ethnology and Sociology, Minzu University of China, Beijing, China; 2 Rory Meyers College of Nursing, New York University, New York, USA; 3 Department of Global Health and Social Medicine, King’s College London, London, WC2R 2LS UK

**Keywords:** Formal home and community-based care, Daily living assistance, Community-based health care, Informal care, Older people

## Abstract

**Background:**

Formal home and community-based care are often considered as the preferable option to institutional care, offering older individuals the convenience of receiving care in their homes. Although research has found that these services may alleviate the burden on informal caregivers, there is a lack of research on which specific types of formal home and community-based care influence informal care provision.

**Methods:**

Employing fixed-effects and quantile regression models, this study seeks to explore the effects that various formal home and community-based care services have on reducing the burden of informal care. This study draws data from the Chinese Longitudinal Healthy Longevity Survey 2005, 2008, 2011, 2014, and 2018.

**Results:**

Our findings indicate that two types of formal care substantially influence the provision of informal care. The availability of daily living assistance services correlates with reduced informal caregiving hours, especially for those with extensive care needs. The availability of community-based health care services is linked to a reduction in the direct expenses incurred from informal caregiving, especially for those incurring greater direct caregiving costs. These effects are more prominent among urban residents. Other services, such as mental health support and legal advice services, do not demonstrate significant effects on reducing informal care hours and costs.

**Conclusions:**

Daily living assistance and community-based health care services play a crucial role in benefiting informal caregivers. It is important to prioritize the expansion of these services, especially among those with greater care needs.

**Supplementary Information:**

The online version contains supplementary material available at 10.1186/s12877-024-05312-7.

## Introduction

‘Ageing in place’ is a commonly used term in ageing policy, defined as ‘helping older people to remain in their homes and communities safely, independently, and comfortably’ [1]. It is favored by policymakers as it is considered as an effective way to curb the rising costs for institutional care [2]. Unpaid informal care, frequently offered by family members and friends, represents the most prevalent form of care for older individuals residing in communities [3]. However, given the declining number of informal caregivers and shifting social norms, relying solely on informal caregivers to provide care is unlikely to be sustainable in the long run [4].

Formal home and community-based care refers to a variety of services offered to individuals with chronic illnesses, or functional limitations, enabling them to receive continuous care and support while ageing in the comfort of their own homes or within their communities, rather than relocating to nursing homes or residential facilities [5]. Formal home and community-based care services initially focus on providing assistance with daily living activities, gradually expanding to include health care services, such as medication management and rehabilitation services [6]. For example, the US and UK initiated community care during the mid to late 20th century, providing daily living assistance services and specialist visits within community settings [6]. Some Asian countries also witnessed the development of formal home and community-based care. For instance, in Japan and Hong Kong, community-based care services have become increasingly important [7, 8]. Programs within the neighborhoods and communities have been launched to include daycare centers, home-based care, and residential care services, all of which enables seniors to maintain an active and involved role within their local communities [7].

The availability of formal home and community-based care has profound effects on the demand of informal care. However, the findings on the effect of formal home and community-based care on informal care are mixed. In terms of daily living assistance, several empirical studies in Europe indicate that services aimed at assisting older people with activities of daily living could help address specific care needs and offer respite to informal caregivers who may be struggling with caregiving demand [9, 10]. Thus, formal home and community-based care providers may emerge as viable alternatives to informal care. Other studies suggest that increased assistance with daily living activities is associated with receiving a greater amount of informal care from adult children [11].

Literature also presented mixed evidence regarding the relationship between community-based health care and informal caregiving. Empirical studies from Sweden and Denmark suggest that a higher frequency of home visits by health workers correlates with a reduced amount of informal caregiving [9, 12]. These health workers often possess professional certifications and undergo extensive training, equipping them with the necessary skills to adeptly address the complex health and care needs of older people. Such health care services have the potential to positively influence the health of older people, thereby decreasing the reliance on informal care [9]. On the contrary, others find a positive relationship between the number of home visits and amount of informal care [10]. One plausible explanation is that certain medical services administered by community-based doctors or nurses are not typically tasks that family caregivers would be anticipated to perform or substitute. Therefore, older individuals rely on informal caregivers to facilitate their access to these specialists.

China also initiated the development of their formal home and community-based care systems. As early as 2000, the Chinese government laid out the strategy to strengthen the community’s role in delivering care services. The concept of formal home and community-based care is officially defined as services provided by the government and social organizations within the community [13]. These services could be classified into four types: daily living assistance (such as assistance with eating, bathing, walking, and household chores), community-based health care (such as home visits from health workers), mental health support (such as emotional comfort), and legal advice services (such as rights protection). It is important to note that, in China, formal home and community-based extends beyond the scope of solely addressing daily living activities in order to address the diverse needs of older individuals. The range of services offered also include various forms of support, including organizing recreational activities, regular check-ins with service recipients, mediating neighborhood disputes and offering human rights counseling to safeguard the rights of older people.

Understanding the relationship between different types of these care services and informal care has significant policy implications in China as the country has witnessed a reduction in informal care provision, attributed to various factors such as urbanization, changing family structures, and increased workforce participation among family members [14]. These shifts are reshaping the landscape of care for older people. Although formal home and community-based care has achieved rapid development and considerable progress in China [15], very little is known on the effect of these services on informal care provision. China provides a broader spectrum of formal home and community-based care services compared to Western countries [16]. Previous empirical studies conducted in Western countries concerning the correlation between formal home and community-based care and informal care predominantly focus on aspects, such as daily living assistance or community-based health care, often neglecting other types of care services that have recently emerged in China. In practice, formal care services exhibit substantial variations in terms of their types, coverage, intended beneficiaries, and the frequency of care delivery. These services may have varying effects on informal caregiving by addressing different needs of older individuals. In addition, there is a dearth of studies to examine different effects of these services across different segment of the populations. For example, older people residing in prosperous urban regions may experience greater advantages from formal home and community-based care, with services being more accessible and of higher quality compared to rural areas [4]. As a result, the influence of formal care on informal care may be more significant in these areas.

Based on the discussion above, this study seeks to examine the relationship between formal home and community-based care and informal care in China. We pay particular attention to different types of home and community-based care on informal care provision, and explore different effects of these services on informal care based on rural-urban residence. We focus our study on those who have functional limitations as these people are the intensive care users and direct beneficiaries from formal and informal care [17]. This study uses data from the Chinese Longitudinal Healthy Longevity Survey (CLHLS) 2005, 2008, 2011, 2014 and 2018 waves. The rest of the paper will discuss relevant literature and the context of China’s formal home and community-based care system, followed by methods, results, discussion and conclusion.

## Formal home and community-based care and informal care

Empirical research on the relationship between formal home and community-based care and informal care yields inconclusive findings. Unlike institutional care, which provides round-the-clock care and services to older individuals, formal home and community-based care is typically provided on a part-time basis. Depending on the specific services offered, older individuals may still necessitate additional care from paid caregivers or their family members.

Research indicates that because informal caregivers, often considered lacking professional skills, frequently assist with basic daily activities, formal home and community-based care can serve as a viable substitute for certain care tasks that demand low-level skills, such as bathing and mobility [18]. Furthermore, additional home and community-based services like grocery shopping and medication delivery may help reduce the time and associated costs borne by informal caregivers.

Some research indicates that community-based health care, such as home visits and health education, may function as substitutes for informal care [9, 12]. Home visits by health care professionals enable regular health monitoring, early detection of health issues, and timely intervention. This proactive approach can help prevent health crises and reduce the needs for informal caregivers to step in during emergencies. Health education provides older people with the knowledge and skills to manage health conditions effectively. Consequently, older people may better care for themselves, thereby reducing their reliance on informal caregivers for basic health-related tasks. Similarly, certain community-based health care services, such as rehabilitation care, can be complementary to complement informal care [19, 20]. Studies propose that some services requiring advanced skills are less likely to be assumed by family members. Instead, older individuals may rely on informal caregivers to facilitate access to these specialized services [21]. For instance, family members often assist older individuals in scheduling appointments, arranging transportation to health care or long-term care providers, and encouraging them to undergo more advanced diagnostic procedures and treatments.

Although mental health support, emotional comfort, and legal aid services within the community is an important part for caring for older people [22], these services are still in the initial stage of the development in most countries and have been largely overlooked by most studies. Some studies conducted in China demonstrate that community-based mental health support, including regular check-ins, emotional comfort and participation in recreational activities, contributes significantly to both physical and mental health improvements among older people [23–25]. Regular check-ins provide emotional comfort to older individuals by allowing them to express their concerns and worries. Involvement in recreational activities strengthens social support networks, fosters relaxation and enjoyment, and consequently reduces stress levels. Older people may then require less assistance from informal caregivers to manage emotional distress or mental health concerns. In terms of legal advice services, including legal aid and handling neighborhood disputes, different findings exist regarding their effect on the health of older people. While some studies suggest that these services do not have significant effects on older people’s health [24, 25], others indicate that legal aid aimed at promoting and protecting the rights and dignity of older people can positively influence their health outcomes [26]. The latter studies propose that legal aid can facilitate access to essential services such as health care and social welfare benefits for older people. By advocating for their rights to receive these services, legal aid can contribute to maintaining their independence and enhancing their quality of life, which may reduce their dependence on informal caregivers. However, there remains a shortage of empirical studies examining the relationship between these types of formal home and community-based care and informal care.

Another significant variable that could add complexity to the entire situation is the regional disparities in the care provision [19, 27]. Specifically, the local government structures initiatives to provide daily living assistance, basic health assessments, and other types of services to individuals requiring support within a community or home-based context. Such endeavors necessitate substantial financial commitments from local government. When comparing regions with stronger economic development to less-developed areas, it becomes evident that the former has higher government revenue and more substantial financial resources for the development of formal care services. Consequently, these disparities can result in variations in the availability and quality of care provided through publicly funded formal care services within each region. This argument also applies to China, where noticeable regional disparities, particularly in rural-urban inequalities, are prevalent. The expansion of formal home and community-based care is highly uneven between these settings. By 2022, these services had covered most urban areas, but only extended to half of the rural areas [28]. Inequalities are also found in the training and skills of care providers. In poor rural areas, care providers lack professional training and skills, whereas in large cities, care providers receive regular training to provide high-quality services, and older people in urban areas have greater access to high-quality community care than those in rural areas [4]. What remains unclear is how these disparities influence the relationship between formal home and community-based care and informal care, as well as their potential implications for older individuals.

## The case study of China

This study focuses on China, where formal home and community-care is thriving since 2000. The concept of formal home and community-based care is officially defined as services provided by the government and social organizations within the community [13]. These services include daily living assistance (such as assistance with eating, bathing, walking, and household chores), community-based health care (such as home visits from health workers), mental health support (such as emotional comfort), and legal advice services (such as rights protection). In 2001, the Ministry of Civil Affairs initiated the Starlight Project to improve community-based services specifically for older people. This project aimed to provide a wide range of services tailored to the needs of older people, including physical and leisure activities, health promotion workshops, and various forms of care. These services were implemented on a nationwide scale, with the goal of enhancing the well-being and quality of life of older people across the country [29]. By 2005, China established 32,000 Starlight daycare centers across the country [30]. Over time, the Starlight Project encountered various challenges, partly attributed to reduced financial support from the government. In response, in 2016, the central government introduced a pilot program aimed at mitigating both funding shortages and the issue of unsustainable funding. The number of pilot regions expanded from 26 in 2016 to 203 in 2021, covering all provinces across mainland China [28]. With the implementation of these initiatives, formal home and community-based care has been recognized as a necessary approach to supporting older people in ageing at home in government’s strategic plan [4].

These services can be categorized into home-based services and center-based services based on the target population [13]. Home-based services are designed to meet daily living and basic health care needs of individuals with functional limitations. These services include personal care, household assistance, home visits, and health education. Centre-based services are designed to meet diverse needs of a larger population, including those with and without functional limitations. These services extend from daily living assistance and basic health care to include counseling, social and recreational activities, and other educational developmental activities. The majority of service users are expected to be responsible for the costs of care. The government steps in only when older people have no other resources, such as family caregivers and pension income. In practice, this limited support is reflected in the stringent eligibility criteria for government support. Financial support from the government is restricted to older people with disabilities and low incomes, and those with minimal informal care support, subject to evaluation and validation by third-party specialists. Varying eligibility criteria may be implemented across different regions to assess needs [4]. In addition, since different regions vary markedly in terms of economic development, there is a great divide in care availability: these formal care services are mainly offered in provincial capitals or large cities, whereas they are not prevalent in poor rural counties and villages [30]. Table 1 gives an overview of key features of China’s formal home and community-based care.

**Table 1:** Overview of key features of formal home and community-based care in China

Evolving policy	In 2000, the State council laid out the strategy to develop home and community-based care.	
	In 2008, the National Working Commission on Ageing along with other departments jointly defined ‘home and community-based care’.	
	In 2011, the central government planned to build a social care system where ‘home care is the foundation, community care provides the necessary support, and residential care is supplementary’. The local governments interpreted this plan into the ‘9073’ and ‘9064’ model (i.e., 90% of older people live independently in their own homes or receive home care, 7%/6% receive community care, and the rest (3%/4%) live in nursing homes).	
	In 2016, the central government launched a pilot program for formal home and community-based care in 26 regions.	
	In 2021, the number of pilot regions increased to 203, covering all provinces throughout mainland China.	
Type of service provided	Home-based services Delivered at home	Services: personal care, daily shopping assistance, rehabilitation exercises, care management, basic and special health care, counselling services, 24-hour emergency support, environmental risk assessment, home modifications, cooking and meal delivery services, transportation and attendant services
	Centre-based services Delivered in day care centres or neighbourhood community centres	Services: counselling services, educational and developmental activities, provision of information on community resources and referral services, reaching out and networking, meal and laundry services, drop-in service, social and recreational activities, caregiver support services
Availability	By 2022, formal home and community-based care services have covered most cities, but only extended to half of the rural areas. Personal care, household assistance, basic Community-based health care are the main types of services, while the availability of rehabilitation services and emotional support is relatively limited.	
Accessibility	In some large cities, some day care centres are located within an approximate 15-minute walking distance range, while in some poor rural areas, there is no day care centre within a village.	
Quality	In some large cities, care providers receive regular training to provide high-quality services, while in some rural areas, care providers lack professional training and skills.	
Affordability	The cost of formal home and community-based care services is partially subsidised by the local governments, and the level of support for care recipients varies among different regions. In some provinces, government support is only available to older people in urban areas who meet a specific age requirement and have moderate/severe functional limitations.	

Source: Hu et al. (2018); Huang and Sun (2021); Su et al. (2023)

Despite the considerable development of formal home and community-based care in China following these initiatives, the relationship between these services and informal care remains poorly investigated. Therefore, this study aims to investigate the effect of various types of formal home and community-based care on informal care. Additionally, we will examine the distinct effects of these services on informal care based on rural-urban residence. Given the central government’s prioritization of the development of formal home and community-based care services to alleviate the burden on family caregivers and mitigate the urban-rural disparity in care provision [16], the findings of this study may help policymakers in evaluating the efficacy of the policy. Specifically, it can provide insights into whether the promotion of formal home and community-based care effectively lessens the burden on informal caregivers and whether rural-urban disparities in care services persist.

## Data and methods

### Data and sample

This study uses the data from the 2005, 2008, 2011, 2014, and 2018 waves of the CLHLS, a nationally representative survey of healthy longevity in China. It was approved by the Research Ethics Committee of Peking University (IRB00001052-13074), and all participants or their proxy respondents provided written informed consent. Initiated in 1998, the CLHLS is to investigate the determinants of older people’s health and longevity in China from a multidisciplinary perspective. The sampling design adopts a multi-stage disproportionate and targeted random sampling method. It has the largest sample of the oldest old (aged 80 and over) in China, who are most likely to need health and social care, and a comparable sample of younger older people (aged 65–79). It provides information on demographic, family structure, living arrangements, self-rated health, chronic disease, care needs and costs, psychological characteristics, socioeconomic status, family support, daily living activities, and cognitive function. Many researchers have formally registered as CLHLS data users, and numerous papers using this survey have been published in international peer-reviewed journals that focus on health care and long-term care [31, 32].

In the CLHLS survey, the question on informal care is a three-tier question. Respondents were first asked whether they required assistance in carrying out each activity of daily living (ADL), including eating, dressing, indoor mobility, bathing, toileting, and continence. If they reported that they did, they were then asked to choose one of the answers below as their primary caregiver: spouse, children, grandchildren, other relatives, friends, neighbors, social services, or housekeepers. Based on previous studies, spouse, children, grandchildren, other relatives, friends, and neighbors are identified as the informal caregivers [14, 31, 32]. If the respondent selected one of them, additional questions were then posed regarding the total number of hours they received help in the last week and overall direct costs associated with informal care. Therefore, our analysis is restricted to individuals aged 65 and above with at least one ADL limitation, who primarily rely on informal care, and who have participated in at least two waves of the survey conducted in 2005, 2008, 2011, 2014, and 2018. This final unbalanced panel data sample comprises 12,514 participants, distributed across the waves as follows: 3,037 participants in 2005, 2,971 in 2008, 2,340 in 2011, 1,757 in 2014, and 2,409 in 2018. Descriptive statistics of the study sample are presented in Table 2.

**Table 2:** Descriptive statistics of the sample

Variables	Mean (SD)/ Percentages
**Dependent variables**	
Hours of informal care in the last week	64.04 (62.49)
Total direct costs associated with informal care in the last week	186.06 (480.83)
**Independent variables**	
Availability of daily living assistance in the community	
No	90.86
Yes	9.14
Availability of community-based health care in the community	
No	65.76
Yes	34.24
Availability of mental health support in the community	
No	81.91
Yes	18.09
Availability of legal advice services in the community	
No	77.19
Yes	22.81
**Covariates**	
Residence	
Rural	50.40
Urban	49.60
Age	94.58 (8.86)
Gender	
Female	68.02
Male	31.98
Self-rated health	
Bad	44.77
Fair	27.61
Good	27.62
Number of ADL limitations	2.85 (1.87)
Cognitive function	13.73 (10.61)
Whether suffering from hypertension	
No	74.29
Yes	25.71
Whether suffering from heart disease	
No	85.73
Yes	14.27
Whether suffering from cardiovascular disease	
No	85.96
Yes	14.04
Whether suffering from diabetes	
No	95.49
Yes	4.51
Household per capita income	16108.05 (21177.78)
Education level	
Illiteracy	86.99
Elementary school and above	13.01
Marital status	
Married	16.15
Other	83.85
Living arrangement	
Living Alone	7.75
Living with spouse	15.89
Living with other family members	76.36
Having social health insurance	
No	35.29
Yes	64.71
Having old age pension	
No	70.20
Yes	29.80
Number of surviving adult children	3.55 (1.94)
Money transfers received from children	2556.91 (4940.84)
N	12,514

*Notes* The unit of this study sample is the individual. These characteristics are the summary statistics across waves. Mean (SD) is presented for continuous variables, and Percentages is presented for categorical variables. ADL = activity of daily living

### Variable specification

#### Dependent variables

The outcome of interest is informal care, including hours of informal care and direct costs associated with informal care. The CLHLS collected this information by asking ‘How many hours in total did your children, grandchildren and their spouses help you in ADLs last week’, and ‘How much is the total direct costs last week paid for primary care, including expenses related to transportation, medical supplies, and additional household needs’. Bottom and top 0.5% of distributions of informal care hours and costs are trimmed following the convention [33]. In all models, we logarithmically transform total direct costs to account for non-linearities. The measurement of informal care hours and associated costs are frequently used in previous studies [14, 31, 32]. However, it is crucial to recognize the limitation in clearly distinguishing between different types of informal care in many surveys. While some informal care primarily focuses on daily living assistance, it also includes additional assistance, such as medication delivery, emotional comfort, regular chat, and mediation of neighborhood conflicts. Given that certain types of informal care may be provided simultaneously, distinguishing them distinctly becomes challenging. Most studies treat all types of informal care collectively in analysis, making it difficult to examine the relationship between different types of formal care and various forms of informal care.

#### Independent variables

We construct four binary independent variables to indicate different types of formal home and community-based care services, i.e., daily living assistance, community-based health care, mental health support, and legal advice services. These variables are derived from the below question ‘What kind of long-term care services are available in your community?’. This question included eight specific items, including personal daily care services, home visits and medication delivery, emotional comfort and regular chat, daily shopping, social and recreation activities, legal assistance (rights protection), health education, and handling neighborhood disputes. We follow the approach adopted by previous studies [23, 24], and classify these items into four types. In particular, daily living assistance includes personal daily care services and daily shopping; community-based health care includes home visits from healthcare professionals and medication delivery, and health education; mental health support includes emotional comfort and regular check-ins, as well as social and recreation activities; legal advice services include legal assistance (rights protection) and handling neighborhood disputes. The binary variable is denoted as 1 if one or more services are available in the respondents’ communities and 0 otherwise. For example, in the binary variable of daily living assistance, 1 represents the availability of personal daily care services or/and daily shopping.

#### Covariates

The key variable in this study is the current residential status of the respondents, categorized into two groups: rural areas (the reference group) and urban areas. Based on relevant studies [23, 24], we control for a set of needs-related variables, including age, gender, self-rated health, number of ADL limitations, cognitive function, whether suffering from hypertension, whether suffering from heart disease, whether suffering from cardiovascular disease, and whether suffering from diabetes. Age is a continuous variable measured by years. Gender is a binary variable with the female set as the reference category. Self-rated health is a categorical variable, comprising ‘bad’ (the reference group), ‘fair’ and ‘good’ status. Number of ADL limitations is measured based on the number of the six ADLs the respondent is unable to perform or experience some difficulties with. Cognitive function score is a count variable measured using the Chinese Mini-Mental State Examination. The validity and reliability of this examination has been verified in many studies [34]. The questionnaire had 24 items on orientation, reaction, calculation, recall, and language. The total score was 30 with higher scores indicating better cognitive function. We construct a single score which is then normalized [35]. Based on previous studies [36], we control for several prevalent chronic diseases in China, including hypertension, heart disease, cardiovascular disease, and diabetes. Each of these variables is binary, with ‘Not suffering from this disease’ serving as the reference category.

Socioeconomic-related variables are also included in the analysis: household per capita income, educational level, marital status, living arrangement, number of surviving adult children, money transfers received from children, having social health insurance, having old age insurance. Household per capita income is a continuous variable measured by the question, ‘What was the income per capita of your household last year’. Income in 2005, 2008, 2011, and 2014 are inflated to 2018 values using Consumer Price Indexes. Household size and demographic composition are taken into consideration to adjust household income using the Equivalent Scale [37]. In all models, we use the natural logarithmic of household income to account for non-linearities [38]. Educational level has two categories: illiteracy (the reference group) and elementary school and above. Marital status has two categories: married (the reference group), and other. Living arrangement is a categorical variable, consisting of three categories: ‘living alone’ (the reference group), ‘living with spouse’, and ‘living with other family members’. Number of surviving adult children is a discrete variable for the number of surviving children the respondent has. Money transfers received from children is a continuous variable measured by the question, ‘How much money (including cash and value of materials) did you get last year from your children and their spouse’. Using Consumer Price Indexes, money transfers in 2005, 2008, 2011, and 2014 are inflation-adjusted to 2018 values. We logarithmically transform this variable. Having social health insurance is a binary variable with ‘no’ set as the reference category. Having old age insurance is a binary variable with ‘no’ set as the reference category.

### Empirical strategy

Fixed effects (FE) panel data regression model is used to examine the effect of availability of formal home and community-based care on informal care. In essence, FE model uses individuals as their own control, by comparing their informal care outcomes when exposed to a given level of the availability of formal home and community-based care with their own informal care outcomes when they are exposed to a different level of availability. Assuming that intra-individual changes in exposure are uncorrelated with changes in other variables, the difference in informal care outcomes between the waves is an estimate of the association between formal home and community-based care and informal care for that individual [39]. Averaging these differences across all individuals in the sample yields an estimate of the ‘average treatment effect’, which controls for all time-invariant individual variables. The specification of our model is as below:

1 $$\:{IFC}_{it}={\alpha\:}_{0}+{\alpha\:}_{1}{HACC}_{i,t}+{\beta\:}_{k}{X}_{i,t}^{k}+{\delta\:}_{i}+{{\epsilon\:}}_{\text{i}\text{t}}$$

$\:{IFC}_{it}\:$denotes hours of informal care or direct costs associated with informal care for an individual $\:i$ at wave $\:t$. $\:{HACC}_{i,t}$ denotes the availability of daily living assistance, community-based health care, mental health support, or legal advice services in the community for an individual $\:i$ at wave $\:t$. $\:{X}_{i,t}^{k}$ denotes all covariates for an individual $\:i$ at wave $\:t$. $\:{\alpha\:}_{1}\:$denotes the relationship between availability of formal home and community-based care and informal care. It represents the average change in the dependent variable $\:{IFC}_{it}$ when the binary independent variable $\:{HACC}_{i,t}$ changes from 0 to 1, controlling for covariates and all time-invariant individual-specific characteristics.$\:\:{\delta\:}_{i}\:$ denotes the individual-level unobserved heterogeneity. $\:{{\epsilon\:}}_{\text{i}\text{t}}$ is the error term.

Linear regression may ignore heterogeneity in the relationship of interest, leaving out important variations across the distribution of outcome variable, and masking potential inequalities within the data [40]. Therefore, quantile regression with FE model is employed to find the relationship of interest across the distribution of informal care. The $\:\theta\:$-th conditional quantile function of the outcome variable is specified as follows:

2 $$\:{{Q}_{\theta\:}(IFC}_{it})={\alpha\:}_{0}(\theta\:)+{\alpha\:}_{1}(\theta\:){HACC}_{i,t}+{{\beta\:}_{k}\left(\theta\:\right)X}_{i,t}^{k}+{\delta\:}_{i}+{{\epsilon\:}}_{\text{i}\text{t}}$$

The denotations of $\:{IFC}_{it}\:$,$\:\:{HACC}_{i,t}$, $\:{X}_{i,t}^{k}$, $\:{\delta\:}_{i}\:$, and $\:{{\epsilon\:}}_{\text{i}\text{t}}$ are the same as in Eq. 1. $\:{\alpha\:}_{1}\left(\theta\:\right)$ denote the coefficient of the effect of formal home and community-based care on informal care in relation to the $\:\theta\:$-th quantile function. In this study, we report the regression results for the 10th, 30th, 50th (median), 70th, and 90th quantiles. Subgroup analysis is then performed by rural-urban areas.

## Results

Table 3 shows the association between the availability of formal home and community-based care and hours of informal care using FE model. The availability of daily living assistance significantly reduces hours of informal care received, whereas the availability of the other three types of formal home and community-based care shows no significant correlation with the hours of informal care received. In particular, Model 1 shows that the average change in hours of informal care received last week is reduced by 14.94 h when the binary independent variable (i.e., the availability of daily living assistance) changes from 0 to 1, controlling for covariates and all time-invariant individual-specific characteristics ($\:p$<0.05). Specifically, living in communities with daily living assistance is associated with an average reduction of 14.94 h of informal care received during the past week. Model 2, 3, and 4 indicate that the availability of community-based health care, mental health support, and legal advice services does not have significant effect on the hours of informal care received. In Model 5, after including these four types of services into analysis, the negative effect of the availability of daily living assistance on informal care remains statistically significant ($\:\beta\:$=-13.970, $\:p$<0.05).

**Table 3:** Association between the availability of formal home and community-based care and hours of informal care

Variables	Model 1	Model 2	Model 3	Model 4	Model 5
**Daily living assistance (Reference: No)**					
**Yes**	-14.943 (6.210) **				-13.970 (6.719) **
**Community-based health care (Reference: No)**					
**Yes**		2.611 (4.047)			4.492 (4.458)
**Mental health support (Reference: No)**					
**Yes**			-7.816 (4.659)		-4.871 (5.213)
**Legal advice services (Reference: No)**					
**Yes**				0.773 (4.168)	3.474 (4.655)
Age	5.224 (3.384)	5.418 (3.386)	5.471 (3.388)	5.126 (3.484)	5.107 (3.487)
Marital status (Reference: Married)					
Other	1.610 (8.953)	1.596 (8.965)	3.837 (9.060)	0.970 (9.083)	2.758 (9.159)
Household per capita income	0.152 (1.231)	0.234 (1.232)	0.173 (1.234)	0.265 (1.239)	0.104 (1.239)
Money transfers received from children	-1.289 (0.542) **	-1.312 (0.542) **	-1.235 (0.541) **	-1.240 (0.546) **	-1.317 (0.548) **
Residence (Reference: Rural)					
Urban	-2.211 (4.338)	-1.644 (4.342)	-1.266 (4.361)	-1.203 (4.373)	-1.506 (4.387)
Living arrangement (Reference: Living alone)					
Living with spouse	5.939 (16.590)	4.205 (16.491)	13.365 (13.365)	4.851 (16.717)	15.245 (17.302)
Living with other family members	-8.134 (8.398)	-9.211 (8.359)	-8.539 (8.364)	-9.469 (8.412)	-9.117 (8.445)
Number of surviving children	-1.589 (1.983)	-1.422 (1.983)	-1.451 (1.978)	-1.349 (1.986)	-1.489 (1.995)
Having social health insurance (Reference: No)					
Yes	-0.090 (4.213)	0.466 (4.229)	-0.053 (4.218)	0.842 (4.285)	0.113 (4.291)
Having old age pension (Reference: No)					
Yes	2.442 (4.947)	1.014 (4.923)	1.444 (4.934)	1.631 (4.977)	2.972 (5.005)
Self-rated health (Reference: Bad)					
Fair	-0.138 (4.267)	-1.406 (4.282)	-1.171 (4.285)	-1.155 (4.301)	-0.701 (4.332)
Good	-3.258 (4.477)	-4.116 (4.497)	-3.850 (4.502)	-4.207 (4.528)	-3.457 (4.548)
Number of ADL limitations	9.013 (1.126) ***	8.761 (1.125) ***	8.824 (1.127) ***	8.608 (1.132) ***	8.857 (1.140) ***
Cognitive function	-0.531 (0.222) **	-0.577 (0.222) ***	-0.562 (0.222) **	-0.581 (0.223) ***	-0.536 (0.225) **
Whether suffering from hypertension (Reference: No)					
Yes	4.524 (4.915)	5.049 (4.913)	5.760 (4.930)	5.618 (4.945)	4.546 (4.950)
Whether suffering from heart disease (Reference: No)					
Yes	-4.794 (5.895)	-3.924 (5.899)	-3.881 (5.893)	-5.130 (5.950)	-5.979 (5.947)
Whether suffering from cardiovascular disease (Reference: No)					
Yes	3.366 (6.003)	3.171 (6.025)	3.594 (6.047)	4.012 (6.060)	3.109 (6.120)
Whether suffering from diabetes (Reference: No)					
Yes	-4.316 (12.808)	-5.017 (12.673)	-5.249 (12.680)	-7.770 (12.825)	-6.161 (12.971)
Year (Reference: 2005)					
2008	-7.487 (11.859)	-9.398 (11.850)	-9.252 (11.873)	-7.979 (12.176)	-6.553 (12.205)
2011	-23.302 (21.696)	-25.317 (21.700)	-24.883 (21.721)	-23.507 (22.316)	-23.326 (22.355)
2014	-33.075 (30.694)	-36.358 (30.676)	-35.989 (30.710)	-34.000 (31.566)	-32.960 (31.625)
2018	-25.148 (44.699)	-27.761 (44.699)	-28.891 (44.741)	-25.556 (45.844)	-23.853 (45.888)
N	12,514				

*Notes* ADL = activities of daily living. Gender and Education are time-invariant variables, which is omitted in FE models. Cells represent coefficient (robust standard errors). *** *p* < 0.01, ** *p* < 0.05, * *p* < 0.1

Table 4 shows the association between the availability of formal home and community-based care and total direct costs associated with informal care using FE model. The availability of community-based health care significantly reduces the total direct costs associated with informal care, while the availability of the other three types of formal home and community-based care does not show significant correlation with these costs. Specifically, Model 1 shows that the average change in total direct costs related to informal care is reduced by 0.37 units when the binary independent variable (i.e., the availability of community-based health care) changes from 0 to 1, controlling for covariates and all time-invariant individual-specific characteristics ($\:p$<0.05). In particular, living in communities with community-based health care is associated with an average reduction of 0.37 units in total direct costs related to informal care. In Model 5, even after including four types of services into analysis, this negative association remains statistically significant ($\:\beta\:$=-0.325, $\:p$<0.05). Model 2, 3, 4, and 5 indicate that the availability of daily living assistance, mental health support, and legal advice services does not have significant effect on total direct costs associated with informal care.

**Table 4:** Association between the availability of formal home and community-based care and total direct costs associated with informal care

Variables	Model 1	Model 2	Model 3	Model 4	Model 5
**Daily living assistance (Reference: No)**					
**Yes**	-0.219 (0.268)				-0.162 (0.290)
**Community-based health care (Reference: No)**					
**Yes**		-0.371 (0.174) **			-0.325 (0.169) **
**Mental health support (Reference: No)**					
**Yes**			-0.351 (0.201) *		-0.217 (0.227)
**Legal advice services (Reference: No)**					
**Yes**				-0.083 (0.178)	0.065 (0.201)
Age	0.291 (0.142) **	0.291 (0.142) **	0.282 (0.141) **	0.283 (0.146) *	0.292 (0.143) **
Marital status (Reference: Married)					
Other	-0.364 (0.414)	-0.370 (0.414)	-0.469 (0.419)	-0.350 (0.417)	-0.385 (0.414)
Household per capita income	-0.011 (0.057)	0.001 (0.056)	-0.011 (0.056)	-0.002 (0.057)	-0.007 (0.057)
Money transfers received from children	0.022 (0.025)	0.024 (0.024)	0.025 (0.024)	0.028 (0.024)	0.024 (0.025)
Residence (Reference: Rural)					
Urban	0.019 (0.190)	-0.005 (0.190)	-0.004 (0.189)	0.016 (0.204)	0.001 (0.190)
Living arrangement (Reference: Living alone)					
Living with spouse	0.885 (0.720)	0.812 (0.710)	0.747 (0.749)	0.987 (0.723)	0.759 (0.751)
Living with other family members	0.385 (0.379)	0.492 (0.376)	0.492 (0.376)	0.549 (0.379)	0.465 (0.381)
Number of surviving children	-0.056 (0.084)	-0.052 (0.349)	-0.032 (0.083)	-0.042 (0.084)	-0.058 (0.084)
Having social health insurance (Reference: No)					
Yes	-0.188 (0.182)	-0.162 (0.182)	-0.191 (0.182)	-0.170 (0.185)	-0.174 (0.183)
Having old age pension (Reference: No)					
Yes	0.015 (0.221)	-0.002 (0.219)	-0.027 (0.218)	-0.022 (0.221)	0.037 (0.221)
Self-rated health (Reference: Bad)					
Fair	-0.371 (0.188) **	-0.340 (0.190) *	-0.356 (0.188) *	-0.404 (0.190) **	-0.334 (0.190)
Good	-0.247 (0.193)	-0.256 (0.194)	-0.231 (0.193)	-0.269 (0.195)	-0.334 (0.190) *
Number of ADL limitations	0.307 (0.048) ***	0.316 (0.048) ***	0.308 (0.048) ***	0.301 (0.048) ***	0.315 (0.048) ***
Cognitive function	0.009 (0.009)	0.012 (0.010)	0.011 (0.009)	0.009 (0.009)	0.010 (0.010)
Whether suffering from hypertension (Reference: No)					
Yes	0.360 (0.215) *	0.386 (0.215) *	0.354 (0.216)	0.375 (0.217) *	0.384 (0.217) *
Whether suffering from heart disease (Reference: No)					
Yes	-0.366 (0.259)	-0.316 (0.259)	-0.310 (0.258)	-0.349 (0.261)	-0.382 (0.261)
Whether suffering from cardiovascular disease (Reference: No)					
Yes	0.179 (0.263)	0.175 (0.264)	0.155 (0.265)	0.158 (0.266)	0.114 (0.268)
Whether suffering from diabetes (Reference: No)					
Yes	0.301 (0.532)	0.203 (0.525)	0.150 (0.524)	0.198 (0.525)	0.315 (0.533)
Year (Reference: 2005)					
2008	0.230 (0.502)	0.195 (0.501)	0.277 (0.500)	0.241 (0.516)	0.199 (0.502)
2011	0.196 (0.917)	0.244 (0.918)	0.294 (0.915)	0.207 (0.946)	0.264 (0.920)
2014	-0.677 (1.294)	-0.650 (1.292)	-0.527 (1.289)	-0.702 (1.333)	-0.615 (1.296)
2018	-1.551 (1.878)	-1.499 (1.877)	-1.314 (1.872)	-1.497 (1.927)	-1.479 (1.881)
N	12,514				

*Notes* Total direct costs include expenses related to transportation, medical supplies, and additional household needs. ADL = activity of daily living. Gender and Education are time-invariant variables, which is omitted in FE models. Cells represent coefficient (robust standard errors). *** *p* < 0.01, ** *p* < 0.05, * *p* < 0.1

Table 5 presents the results of quantile regression which explores whether our main results as shown in Tables 3 and 4 vary across quantiles of informal care hours and costs. In particular, among those who received higher hours of informal care, the average change in hours of informal care received last week is reduced by between 13.56 and 16.75 h when the binary independent variable (i.e., the availability of daily living assistance) changes from 0 to 1 ($\:p$<0.05). Among those who incurred higher care costs, the average change in total direct costs related to informal care is reduced by between 0.38 and 0.41 units when the binary independent variable (i.e., the availability of community-based health care) changes from 0 to 1 ($\:p$<0.05). The results suggest that the availability of formal home and community-based services have more pronounced effects on those individuals who have greater care hours or incurring higher total direct informal care costs. The regression results of the full models with covariates are presented in Supplementary Tables 1–2.

**Table 5:** Association between the availability of formal home and community-based care and informal care: quantile regression with FE models

**Variables**	**Hours of informal care**				
	**0.1**	**0.3**	**0.5**	**0.7**	**0.9**
**Daily living assistance (Reference: No)**					
Yes	-11.370 (11.250)	-11.570 (10.760)	-13.563 (6.325) **	-16.478 (5.744) ***	-16.754 (6.225) ***
**Community-based health care (Reference: No)**					
Yes	4.056 (7.927)	4.101 (7.582)	4.555 (4.452)	5.218 (4.047)	5.281 (4.386)
**Mental health support (Reference: No)**					
Yes	-4.037 (8.723)	-4.125 (8.344)	-5.003 (4.901)	-6.287 (4.454)	-6.409 (4.827)
**Legal advice services (Reference: No)**					
Yes	1.718 (8.716)	1.842 (8.337)	3.085 (4.898)	4.901 (4.450)	5.073 (4.823)
	**Total direct costs associated with informal care**				
**Variables**	**0.1**	**0.3**	**0.5**	**0.7**	**0.9**
**Daily living assistance (Reference: No)**					
**Yes**	-0.120 (0.489)	-0.123 (0.469)	-0.162 (0.233)	-0.200 (0.240)	-0.203 (0.252)
**Community-based health care (Reference: No)**					
Yes	-0.335 (0.303)	-0.337 (0.291)	-0.376 (0.137) ***	-0.405 (0.157) ***	-0.407 (0.165) **
**Mental health support (Reference: No)**					
Yes	-0.258 (0.395)	-0.255 (0.379)	-0.217 (0.188)	-0.180 (0.193)	-0.177 (0.204)
**Legal advice services (Reference: No)**					
Yes	0.051 (0.312)	0.052 (0.300)	0.065 (0.148)	0.078 (0.153)	0.079 (0.161)
**N**	12,514				

*Notes* Total direct costs include expenses related to transportation, medical supplies, and additional household needs. All the models control for needs-related variables and socioeconomic-related variables. Full results are presented in Supplementary Tables 1–2. Cells represent coefficient (robust standard errors). *** *p* < 0.01, ** *p* < 0.05, * *p* < 0.1

Table 6 presents the subgroup analysis by residence. The negative effect of the availability of daily living assistance on hours of informal care, as well as the negative effect of the availability of community-based health care on total direct costs of informal care, are significant among urban residents, but not among rural residents. Specifically, among urban residents, the average change in hours of informal care received last week is reduced by 20.27 h when the binary independent variable (i.e., the availability of daily living assistance) changes from 0 to 1 ($\:p$<0.05). This implies that among urban residents, living in communities with daily living assistance is associated with an average reduction of 20.27 h of informal care received during the past week. Similarly, among urban residents, the average change in total direct costs related to informal care is reduced by 0.77 units when the binary independent variable (i.e., the availability of community-based health care) changes from 0 to 1 ($\:p$<0.05). This indicates that among urban residents, living in communities with community-based health care is associated with an average reduction of 0.77 units in total direct costs related to informal care. These negative effects are more pronounced at the higher quantiles ( > = 0.5). The regression results of the full models with covariates are presented in Supplementary Tables 3–7.

**Table 6:** Subgroup analysis in the association between availability of formal home and community-based care and informal care

**Variables**	**Hours of informal care**					**Total rural/urban sample**
	**0.1**	**0.3**	**0.5**	**0.7**	**0.9**	
**Rural**						
**Daily living assistance (Reference: No)**						
Yes	-17.498 (14.547)	-17.512 (14.170)	-17.755 (10.258)	-18.123 (9.224)	-18.145 (10.626)	-17.816 (11.503)
**Community-based health care (Reference: No)**						
Yes	4.042 (10.806)	4.064 (10.526)	4.442 (6.135)	5.016 (5.366)	5.050 (5.665)	4.538 (7.256)
**Mental health support (Reference: No)**						
Yes	-6.446 (13.967)	-6.404 (13.605)	-5.688 (7.930)	-4.601 (6.935)	-4.537 (7.322)	-5.506 (9.647)
**Legal advice services (Reference: No)**						
Yes	7.339 (12.035)	7.351 (11.722)	7.567 (6.832)	7.893 (5.976)	7.912 (6.309)	7.621 (7.510)
**Urban**						
**Daily living assistance (Reference: No)**						
Yes	-17.095 (17.859)	-17.371 (16.969)	-19.733 (10.054) **	-23.217 (9.161) **	-23.607 (10.062) **	-20.273 (10.213) **
**Community-based health care (Reference: No)**						
Yes	2.800 (12.591)	2.870 (11.963)	3.468 (7.081)	4.349 (6.458)	4.448 (7.093)	3.604 (6.745)
**Mental health support (Reference: No)**						
Yes	-3.103 (11.928)	-3.142 (11.333)	-3.479 (6.708)	-3.977 (6.118)	-4.032 (6.720)	-3.557 (7.073)
**Legal advice services (Reference: No)**						
Yes	-6.179 (13.992)	-6.012 (13.294)	-4.578 (7.874)	-2.464 (7.177)	-2.227 (7.883)	-4.251 (7.213)
	**Total direct costs associated with informal care**					
**Variables**	**0.1**	**0.3**	**0.5**	**0.7**	**0.9**	**Total rural/urban sample**
**Rural**						
**Daily living assistance (Reference: No)**						
Yes	-0.083 (0.793)	-0.079 (0.766)	-0.023 (0.362)	0.032 (0.362)	0.035 (0.378)	-0.023 (0.439)
**Community-based health care (Reference: No)**						
Yes	0.057 (0.441)	0.056 (0.426)	0.031 (0.201)	0.005 (0.201)	0.004 (0.210)	0.031 (0.291)
**Mental health support (Reference: No)**						
Yes	-0.675 (0.986)	-0.675 (0.950)	-0.684 (0.389)	-0.685 (0.411)	-0.685 (0.420)	-0.680 (0.401)
**Legal advice services (Reference: No)**						
Yes	0.049 (0.715)	0.049 (0.689)	0.068 (0.249)	0.081 (0.289)	0.081 (0.304)	0.065 (0.302)
**Urban**						
**Daily living assistance (Reference: No)**						
Yes	0.539 (1.106)	0.527 (1.086)	0.393 (0.984)	0.274 (1.124)	0.261 (1.151)	0.401 (0.449)
**Community-based health care (Reference: No)**						
Yes	-0.772 (1.221)	-0.772 (1.162)	-0.773 (0.461) *	-0.773 (0.372) **	-0.773 (0.441) *	-0.773 (0.262) ***
**Mental health support (Reference: No)**						
Yes	-0.143 (0.602)	-0.143 (0.574)	-0.152 (0.334)	-0.161 (0.349)	-0.162 (0.366)	-0.152 (0.318)
**Legal advice services (Reference: No)**						
Yes	0.016 (0.581)	0.016 (0.554)	0.014 (0.323)	0.012 (0.336)	0.012 (0.354)	0.014 (0.340)

*Notes* Total rural sample is 6,307. Total urban sample is 6,207. Total direct costs include expenses related to transportation, medical supplies, and additional household needs. All the models control for needs-related variables and socioeconomic-related variables. Full results are presented in Supplementary Tables 3–7. Cells represent coefficient (robust standard errors). *** *p* < 0.01, ** *p* < 0.05, * *p* < 0.1

We perform three sets of supplementary analysis to examine the robustness of our results. We use the natural logarithmic form of hours of informal care to account for non-linearities (See Supplementary Tables 8–10). Given regional disparities in economic development, there are disparities in the availability of these services across provinces. We merge these longitudinal data with economic data (provincial GDP per capita) from China’s National Bureau of Statistics (Full results can be found in Supplementary Tables 11–13). Lastly, we include number of limitations in instrumental activities of daily living and ‘whether the respondent used health care in a hospital last year’ into analysis to reduce the potential bias (See Supplementary Tables 14–16). The results of these robustness checks are consistent with our main findings.

## Discussion

This study examines the effect of different types of formal home and community-based care on informal care among older people with functional limitations in China. With FE models and quantile regression models, we find that the availability of daily living assistance reduces hours of informal care received, and this negative effect is significantly more pronounced for those receiving more hours of informal care. Similarly, the availability of community-based health care reduces total direct costs associated with informal care received, and this negative effect is significantly more pronounced for those incurring higher direct costs for informal care. These negative effects are more pronounced among urban residents. However, the availability of mental health support and legal advice services does not have significant effect on informal care hours and costs.

Our results are in line with some studies [27]. Daily living assistance in the community, often regarded as low-level skilled care, serves as a substitute for informal care due to its efficacy in addressing care needs. For example, meals-on-wheels services not only support older people in maintaining a nutritious diet but also save time for family caregivers who would otherwise be involved in meal preparation. The availability of grocery delivery services helps older people access to fresh food and curtails the time family members previously spent on shopping trips.

In terms of community-based health care, our results are different from some studies conducted in Western countries [19, 20]. These studies suggest that home visits from healthcare professionals is often complementary to informal care, with an increasing probability of receiving home visits being positively associated with a greater amount of informal care received. Our analysis indicates that the availability of community-based health care reduces the direct costs associated with informal care. One potential explanation is that studies in Western countries primarily focus on the utilization of formal care, whereas our study concentrates on the availability of formal care. The utilization of formal care might be endogenous to informal care, whereas availability is exogenous. The methods employed in studies conducted in Western countries may not fully address the issue of endogeneity, potentially influencing the results. In addition, these studies focus on hours of informal care rather than direct costs associated with informal care due to data limitations, which may also result in different findings. Another possible explanation is that in Western countries, community-based health care services are often provided by trained professionals, such as doctors, nurses, or other health care practitioners. Older individuals may require assistance from caregivers to access community health care centers for advanced treatment. In contrast, in China, these care services are primarily delivered by not-for-profit organizations or volunteers rather than trained professionals [16]. Their capabilities are limited to basic health assessments, such as monitoring blood pressure, medication delivery, medication advice and monitoring. Community health care centers often lack updated facilities, such as poorly maintained fitness equipment and a deficiency in comprehensive rehabilitation and healthcare equipment. Hospital beds in the centers are mainly normal beds. The services they offer can be delivered at home, alleviating the needs for informal caregivers to accompany older individuals to community health care centers. These services may also reduce direct health care costs, such as expenses related to hospital-based basic health assessments, transportation costs incurred from residences to hospitals, as well as medication delivery.

 Our findings show that the availability of mental health support and legal advice services does not have significant effect on informal care hours and costs. One possible explanation for this is underutilization [16]. Despite the availability of these services, they may be underutilized due to factors such as a lack of awareness, adherence to traditional cultural values, or reluctance to seek assistance. Older people may opt to depend on care from family members rather than seeking formal mental health support or legal guidance. They may only seek out these services when confronted with significant challenges. Thus, the effect of availability of these formal care services on informal care is not significant. Furthermore, the relationship between mental health support, legal advice services, and informal care is complex. Factors such as the diverse needs of older people and the involvement of various stakeholders, including care providers, legal experts, and community organizations, may contribute to this complexity. The scarcity of this information may result in an incomplete understanding of the complexities surrounding this issue.

We find significant regional disparity in terms of the effect of formal home- and community care on informal care. This disparity can be attributed to factors such as accessibility, affordability, and preferences. Rural areas are usually situated in remote areas with poor transportation links and infrastructure, driving up higher costs in providing formal care than in urban areas [41]. As a result, the provision of formal home and community-based care in rural areas tends to be more dispersed and distant from older people, in contrast to urban areas where proximity to such services is more common. In addition, urban residents typically possess higher incomes and living standards, increasing the propensity to opt for formal care services, while rural residents often grapple with financial constraints, resulting in lower utilisation of formal care services. Despite the fact that the Chinese government developed long-term care insurance system to promote equitable access to formal care, some pilot cities formulated eligibility criteria that exclude rural residents or provide them with low reimbursement rates and limited covered services [42]. Moreover, the rural areas still cling to the traditional notion of ‘raising children for support during old age’, emphasizing the expectation that the responsibility of caring for older parents should be shouldered by their children. Consequently, the act of paying for formal care continues to carry a stigma within Chinese rural society. Conversely, in rapidly developing urban centers, the influence of modernization has brought about shifts in traditional values. As a result, older people in urban areas have increasingly become receptive to the idea of receiving formal care in the community [43]. Compared to their urban counterparts, older people in rural areas are more inclined to rely on informal care over formal care, resulting in the insignificance of formal care’s effect.

Findings from this study have important policy implications for formal home and community-based care in China. Our findings suggest that the availability of daily living assistance and community-based health care could alleviate the caregiving burden on family members, particularly those who are heavily involved in providing informal care and incurring higher costs associated with it. This underscores the indispensable benefits of these services in supporting family caregivers. However, there exists limited societal recognition of community care workers in China [17]. Many of these workers, often comprising laid-off individuals with minimal education, perform rudimentary and unskilled tasks. Consequently, they are frequently undervalued and subject to prejudice, exacerbated by their meager wages. In locales like Yantai, for instance, their average remuneration falls below the local average [17]. Such societal underappreciation and inadequate compensation present obstacles to the development of formal home and community-based care. Experiences from European countries suggest that the government could collect data on community care workers, including their experiences and needs, to increase public awareness of them [44]. The UK government emphasises that community care workers’ wages should be paid more than the national living wage for full-time employees [45]. Similar measures should be taken in China to improve the working conditions of community care workers.

Our study has some limitations that should be noted. Self-report bias is one limitation. Self-reported subjective measures, such as self-reported availability of formal home and community-based care and self-reported informal care hours and costs, may result in underestimation or overestimation. Moreover, the CLHLS does not clearly differentiate types of informal care. Informal care can be categorized into various major types, including assistance with daily living activities, medication delivery, emotional support, and mediation of neighbor disputes. However, this survey only gathers general information about informal care related to daily assistance, lacking detailed data on hours and costs for each type of informal care. Additionally, different types of care may blend together, making it difficult for respondents to accurately distinguish how much time and money each type of informal care consumes. The hours and costs reported by respondents for daily assistance may also include those related to other forms of informal care. As a result, we are unable to investigate the potential substitution of formal care services for specific types of informal care. Our study represents the initial attempt to examine the effect of formal care on informal care in China; further research is needed to differentiate between types of informal care when information is available. Furthermore, this survey only collects information on hours of care provided by children or grandchildren; it does not collect information on hours of care provided by other sources of care, such as a spouse. We include living arrangement as a control variable in analysis to reduce potential bias stemming from living with a spouse. Future studies are needed to examine how formal home and community-based care affects care received from a spouse other than offspring.

## Conclusion

This study examines the effect of different types of formal home and community-based care on informal care among older people with functional limitations in China. The findings show that the presence of daily living assistance is associated with a reduction in the hours of informal care received, particularly among those who receive higher number of informal care hours. Similarly, the availability of community-based health care is linked to decreased direct costs related to informal caregiving, with a more pronounced effect observed among individuals incurring greater direct caregiving costs. These negative effects are more prominent among urban residents. These findings show that formal home and community-based care services play a crucial role in alleviating the caregiving burden on informal caregivers in China. It is important to prioritise the expansion of such services, while simultaneously addressing disparities in their distribution.

## Electronic supplementary material

Below is the link to the electronic supplementary material.

Supplementary Material 1

## Data Availability

Data are available in a public, open access repository. Researchers can download the datasets free of charge from the following websites: https://opendata.pku.edu.cn; Peking University Open Access Research Database.

## Abbreviations

CLHLS: Chinese Longitudinal Healthy Longevity Survey
ADL: Activity of Daily Living
FE: Fixed-effects model
US: United States
UK: United Kingdom
