# Supplementary material for: Can formal home and community-based care substitute informal care? Evidence from Chinese Longitudinal Healthy Longevity Survey
Source: BMC Geriatr. 2024 Sep 3;24:730. doi: 10.1186/s12877-024-05312-7 (PMC11373235; doi:10.1186/s12877-024-05312-7)
Supplement: Supplementary file 1 — Supplementary Material 1 [file 12877_2024_5312_MOESM1_ESM.docx]

**Supplementary Table 1. Association between the availability of formal home and community-based care and hours of informal care: Quantile regression with FE models**

| **Variables** | **0.1** | **0.3** | **0.5** | **0.7** | **0.9** |
| --- | --- | --- | --- | --- | --- |
| **Daily living assistance (Reference: No)** | |  |  |  |  |
| Yes | -11.370 (11.250) | -11.570 (10.760) | -13.563 (6.325) ** | -16.478 (5.744) *** | -16.754 (6.225) *** |
| **Community-based health care (Reference: No)** | |  |  |  |  |
| Yes | 4.056 (7.927) | 4.101 (7.582) | 4.555 (4.452) | 5.218 (4.047) | 5.281 (4.386) |
| **Mental health support (Reference: No)** | |  |  |  |  |
| Yes | -4.037 (8.723) | -4.125 (8.344) | -5.003 (4.901) | -6.287 (4.454) | -6.409 (4.827) |
| **Legal advice services (Reference: No)** | |  |  |  |  |
| Yes | 1.718 (8.716) | 1.842 (8.337) | 3.085 (4.898) | 4.901 (4.450) | 5.073 (4.823) |
| Age | 5.544 (5.299) | 5.514 (5.090) | 5.198 (3.087) * | 4.705 (2.785) * | 4.665 (2.980) |
| Gender | Omitted | Omitted | Omitted | Omitted | Omitted |
| Education | Omitted | Omitted | Omitted | Omitted | Omitted |
| Marital status (Reference: Married) | | |  |  |  |
| Other | 2.833 (16.970) | 2.854 (16.302) | 3.074 (9.886) | 3.419 (8.919) | 3.447 (9.554) |
| Household per capita income | 0.197 (2.104) | 0.200 (2.021) | 0.229 (1.226) | 0.275 (1.106) | 0.279 (1.183) |
| Money transfers received from children | -1.165 (1.020) | -1.180 (0.980) | -1.332 (0.595) | -1.570 (0.536) *** | -1.589 (0.574) *** |
| Residence (Reference: Rural) | | |  |  |  |
| Urban | -2.693 (7.655) | -2.619 (7.353) | -1.840 (4.461) | -0.626 (4.023) | -0.528 (4.305) |
| Living arrangement (Reference: Living alone) | | | | |  |
| Living with spouse | 16.761 (26.351) | 16.559 (25.205) | 14.532 (14.802) | 11.569 (13.454) | 11.287 (14.582) |
| Living with other family members | -5.867 (15.164) | -6.078 (14.504) | -8.189 (8.523) | -11.276 (7.742) | -11.568 (8.391) |
| Number of surviving children | -1.519 (3.623) | -1.538 (3.480) | -1.737 (2.111) | -2.048 (1.904) | -2.073 (2.037) |
| Having social health insurance (Reference: No) | | |  |  |  |
| Yes | 0.958 (7.593) | 0.885 (7.294) | 0.112 (4.425) | -1.091 (3.990) | -1.189 (4.270) |
| Having old age pension (Reference: No) | | |  |  |  |
| Yes | 3.391 (8.797) | 3.325 (8.450) | 2.632 (5.126) | 1.552 (4.623) | 1.465 (4.947) |
| Self-rated health (Reference: Bad) | | |  |  |  |
| Fair | 2.136 (7.763) | 2.047 (7.457) | 1.103 (4.525) | -0.366 (4.079) | -0.485 (4.365) |
| Good | -1.207 (7.981) | -1.308 (7.666) | -2.369 (4.652) | -4.023 (4.194) | -4.1157 (4.488) |
| Whether suffering from ﻿hypertension (Reference: No) |  |  |  |  |  |
| Yes | 5.214 (8.659) | 5.189 (8.283) | 4.932 (4.863) | 4.557 (4.421) | 4.521 (4.792) |
| Whether suffering from ﻿heart disease (Reference: No) |  |  |  |  |  |
| Yes | -3.250 (10.995) | -3.459 (10.517) | -5.546 (6.184) | -8.597 (5.614) | -8.887 (6.084) |
| Whether suffering from cardiovascular disease (Reference: No) |  |  |  |  |  |
| Yes | -0.453 (10.886) | -0.348 (10.413) | 0.701 (6.116) | 2.236 (5.558) | 2.382 (6.024) |
| Whether suffering from ﻿diabetes (Reference: No) |  |  |  |  |  |
| Yes | -7.208 (22.321) | -7.173 (21.351) | -6.819 (12.535) | -6.302 (11.397) | -6.253 (12.352) |
| Number of ADL limitations | 9.450 (1.223) *** | 8.455 (1.175) | 8.512 (0.712) *** | 8.602 (0.643) *** | 8.609 (0.688) ** |
| Cognitive function | -0.551 (0.413) | -0.545 (0.397) | -0.479 (0.241) ** | -0.376 (0.217) * | -0.367 (0.232) |
| Year (Reference: 2005) | |  |  |  |  |
| 2008 | -9.648 (19.006) | -9.452 (18.257) | -7.375 (11.077) | -4.140 (9.989) | -3.877 (10.689) |
| 2011 | -29.348 (33.875) | -29.049 (32.540) | -25.883 (19.740) | -20.950 (17.803) | -20.549 (19.051) |
| 2014 | -41.142 (47.867) | -40.729 (45.980) | -36.370 (27.893) | -29.579 (25.157) | -29.028 (26.920) |
| 2018 | -33.408 (69.591) | -32.848 (66.849) | -26.944 (40.551) | -17.744 (36.574) | -16.998 (39.137) |
| N | 12,514 | | | | |

Notes: ADL = activities of daily living. ﻿Cells represent ﻿coefficient (robust standard errors). *** p<0.01, ** p<0.05, * p<0.1.

**Supplementary Table 2. Association between the availability of formal home and community-based care and total direct costs associated with informal care: Quantile regression with FE models**

| **Variables** | **0.1** | **0.3** | **0.5** | **0.7** | **0.9** |
| --- | --- | --- | --- | --- | --- |
| **Daily living assistance (Reference: No)** |  |  |  |  |  |
| Yes | -0.120 (0.489) | -0.123 (0.469) | -0.162 (0.233) | -0.200 (0.240) | -0.203 (0.252) |
| **Community-based health care (Reference: No)** | |  |  |  |  |
| Yes | -0.335 (0.303) | -0.337 (0.291) | -0.376 (0.137) *** | -0.405 (0.157) *** | -0.407 (0.165) ** |
| **Mental health support (Reference: No)** |  |  |  |  |  |
| Yes | -0.258 (0.395) | -0.255 (0.379) | -0.217 (0.188) | -0.180 (0.193) | -0.177 (0.204) |
| **Legal advice services (Reference: No)** |  |  |  |  |  |
| Yes | 0.051 (0.312) | 0.052 (0.300) | 0.065 (0.148) | 0.078 (0.153) | 0.079 (0.161) |
| Age | 0.295 (0.242) | 0.296 (0.235) | 0.292 (0.137) ** | 0.289 (0.156) * | 0.289 (0.160) * |
| Gender | Omitted | Omitted | Omitted | Omitted | Omitted |
| Education | Omitted | Omitted | Omitted | Omitted | Omitted |
| Marital status (Reference: Married) | |  |  |  |  |
| Other | -0.278 (0.734) | -0.285 (0.710) | -0.396 (0.414) | -0.484 (0.472) | -0.490 (0.485) |
| Household per capita income | 0.003 (0.085) | 0.002 (0.082) | -0.008 (0.048) | -0.016 (0.054) | -0.016 (0.056) |
| Money transfers received from children | 0.028 (0.044) | 0.028 (0.043) | 0.023 (0.025) | 0.019 (0.028) | 0.020 (0.029) |
| Residence (Reference: Rural) |  |  |  |  |  |
| Urban | -0.052 (0.315) | -0.049 (0.305) | 0.006 (0.178) | 0.050 (0.203) | 0.053 (0.208) |
| Living arrangement (Reference: Living alone) | | | |  |  |
| Living with spouse | 0.721 (1.204) | 0.723 (1.154) | 0.759 (0.573) | 0.794 (0.590) | 0.797 (0.621) |
| Living with other family members | 0.502 (0.659) | 0.499 (0.632) | 0.464 (0.313) | 0.431 (0.323) | 0.429 (0.340) |
| Number of surviving children | -0.060 (0.140) | -0.060 (0.135) | -0.057 (0.078) | -0.056 (0.090) | -0.056 (0.092) |
| Having social health insurance (Reference: No) | |  |  |  |  |
| Yes | -0.205 (0.306) | -0.203 (0.297) | -0.172 (0.173) | -0.146 (0.197) | -0.145 (0.203) |
| Having old age pension (Reference: No) | |  |  |  |  |
| Yes | 0.102 (0.360) | 0.098 (0.348) | 0.031 (0.203) | -0.022 (0.231) | -0.025 (0.238) |
| Self-rated health (Reference: Bad) | |  |  |  |  |
| Fair | -0.355 (0.321) | -0.354 (0.310) | -0.332 (0.181) * | -0.314 (0.206) | -0.313 (0.212) |
| Good | -0.237 (0.330) | -0.236 (0.320) | -0.233 (0.186) | -0.230 (0.212) | -0.230 (0.218) |
| Whether suffering from ﻿hypertension (Reference: No) |  |  |  |  |  |
| Yes | 0.383 (0.364) | 0.383 (0.349) | 0.384 (0.173) ** | -.384 (0.178) ** | 0.384 (0.188) ** |
| Whether suffering from ﻿heart disease (Reference: No) |  |  |  |  |  |
| Yes | -0.362 (0.449) | -0.363 (0.431) | -0.382 (0.214) | -0.400 (0.220) * | -0.402 (0.232) * |
| Whether suffering from cardiovascular disease (Reference: No) |  |  |  |  |  |
| Yes | -0.002 (0.453) | 0.006 (0.434) | 0.115 (0.216) | 0.222 (0.222) | 0.230 (0.234) |
| Whether suffering from ﻿diabetes (Reference: No) |  |  |  |  |  |
| Yes | 0.456 (1.004) | 0.446 (0.963) | 0.313 (0.478) | 0.184 (0.492) | 0.175 (0.518) |
| Number of ADL limitations | 0.323 (0.083) *** | 0.322 (0.080) *** | 0.314 (0.047) *** | 0.307 (0.053) *** | 0.307 (0.055) *** |
| Cognitive function | 0.011 (0.017) | 0.011 (0.016) | 0.010 (0.010) | 0.010 (0.011) | 0.010 (0.011) |
| Year (Reference: 2005) |  |  |  |  |  |
| 2008 | 0.167 (0.841) | 0.169 (0.813) | 0.202 (0.475) | 0.228 (0.541) | 0.230 (0.556) |
| 2011 | 0.248 (1.552) | 0.249 (1.502) | 0.266 (0.876) | 0.279 (0.999) | 0.280 (1.026) |
| 2014 | -0.637 (2.210) | -0.636 (2.138) | -0.613 (1.247) | -0.595 (1.423) | -0.594 (1.460) |
| 2018 | -1.532 (3.185) | -1.529 (3.082) | -1.473 (1.798) | -1.429 (2.050) | -1.426 (2.105) |
| N | 12,514 | | | | |

Notes: Total direct costs include expenses related to transportation, medical supplies, and additional household needs. ADL = activities of daily living. ﻿Cells represent ﻿coefficient (robust standard errors). *** p<0.01, ** p<0.05, * p<0.1.

**Supplementary Table 3. ﻿ Subgroup analysis in the association between availability of formal home and community-based care and informal care**

|  | **Hours of informal care** | | **Total direct costs associated with informal care** | |
| --- | --- | --- | --- | --- |
| **Variables** | **Rural** | **Urban** | **Rural** | **Urban** |
| **Daily living assistance (Reference: No)** |  |  |  |  |
| Yes | -17.816 (11.503) | -20.273 (10.213) ** | -0.023 (0.439) | 0.401 (0.449) |
| **Community-based health care (Reference: No)** | |  |  |  |
| Yes | 4.538 (7.256) | 3.604 (6.745) | 0.031 (0.291) | -0.773 (0.262) *** |
| **Mental health support (Reference: No)** |  |  |  |  |
| Yes | -5.506 (9.647) | -3.557 (7.073) | -0.680 (0.401) | -0.152 (0.318) |
| **Legal advice services (Reference: No)** |  |  |  |  |
| Yes | 7.621 (7.510) | -4.251 (7.213) | 0.065 (0.302) | 0.014 (0.340) |
| Age | 0.625 (7.113) | 5.278 (8.303) | 0.181 (0.256) | 0.299 (0.392) |
| Gender | Omitted | Omitted | Omitted | Omitted |
| Education | Omitted | Omitted | Omitted | Omitted |
| Marital status (Reference: Married) | |  |  |  |
| Other | -1.855 (16.465) | 21.268 (14.013) | -1.648 (0.698) ** | 0.713 (0.646) |
| Household per capita income | 1.365 (1.820) | -1.343 (2.205) | 0.062 (0.077) | -0.195 (0.105) * |
| Money transfers received from children | -2.508 (1.068) ** | -1.556 (0.785) ** | -0.033 (0.044) | 0.016 (0.037) |
| Living arrangement (Reference: Living alone) | | | |  |
| Living with spouse | 37.552 (31.206) | -4.526 (25.787) | -0.014 (1.331) | 1.285 (1.173) |
| Living with other family members | 8.192 (14.430) | -5.613 (13.782) | 0.139 (0.603) | 1.506 (0.680) ** |
| Number of surviving children | -2.754 (3.147) | 2.252 (3.792) | -0.111 (0.124) | -0.056 (0.084) |
| Having social health insurance (Reference: No) | |  |  |  |
| Yes | 4.826 (7.666) | 1.239 (6.493) | -0.273 (0.305) | -0.064 (0.290) |
| Having old age pension (Reference: No) | |  |  |  |
| Yes | 9.889 (9.299) | -1.924 (8.054) | -0.602 (0.386) | 0.098 (0.362) |
| Self-rated health (Reference: Bad) | |  |  |  |
| Fair | 2.904 (7.513) | -11.787 (7.013) * | -0.694 (0.103) ** | -0.572 (0.320) * |
| Good | 0.590 (7.611) | -17.639 (7.686) ** | 0.026 (0.305) | -0.958 (0.338) *** |
| Whether suffering from ﻿hypertension (Reference: No) |  |  |  |  |
| Yes | 4.743 (8.804) | 2.409 (7.803) | 0.637 (0.376) | 0.463 (0.352) |
| Whether suffering from ﻿heart disease (Reference: No) |  |  |  |  |
| Yes | -2.142 (10.738) | -11.879 (8.722) | 0.029 (0.450) | -0.420 (0.411) |
| Whether suffering from ﻿cardiovascular disease (Reference: No) |  |  |  |  |
| Yes | 5.157 (10.734) | 9.482 (9.144) | 0.048 (0.460) | 0.067 (0.436) |
| Whether suffering from ﻿diabetes (Reference: No) |  |  |  |  |
| Yes | 2.976 (30.361) | -3.756 (16.318) | -0.404 (1.141) | 0.120 (0.733) |
| Number of ADL limitations | 8.095 (1.085) | 8.062 (1.105) *** | 0.305 (0.077) *** | 0.281 (0.083) *** |
| Cognitive function | -0.507 (0.384) | -0.203 (0.373) | 0.016 (0.015) | 0.014 (0.016) |
| Year (Reference: 2005) |  |  |  |  |
| 2008 | -3.075 (24.373) | 1.505 (27.391) | 0.322 (0.894) | 0.708 (1.296) |
| 2011 | -10.723 (45.370) | -15.853 (51.580) | 0.957 (1.655) | 0.874 (2.440) |
| 2014 | -18.200 (63.170) | -21.882 (75.044) | 0.086 (2.285) | 0.178 (3.559) |
| 2018 | -16.101 (90.687) | 8.347 (109.956) | 0.502 (3.315) | -1.484 (5.170) |
| N | 6,307 | 6,207 | 6,307 | 6,207 |

Notes: Total rural sample is 6,307. Total urban sample is 6,207. Total direct costs include expenses related to transportation, medical supplies, and additional household needs. Cells represent ﻿coefficient (robust standard errors). *** p<0.01, ** p<0.05, * p<0.1.

**Supplementary Table 4. ﻿ Association between the availability of formal home and community-based care and hours of informal care among rural residents: Quantile regression with FE models**

|  | **Hours of informal care** | | | | |
| --- | --- | --- | --- | --- | --- |
| **Variables** | **0.1** | **0.3** | **0.5** | **0.7** | **0.9** |
| **Daily living assistance (Reference: No)** |  |  |  |  |  |
| Yes | -17.498 (14.547) | -17.512 (14.170) | -17.755 (10.258) | -18.123 (9.224) | -18.145 (10.626) |
| **Community-based health care (Reference: No)** | |  |  |  |  |
| Yes | 4.042 (10.806) | 4.064 (10.526) | 4.442 (6.135) | 5.016 (5.366) | 5.050 (5.665) |
| **Mental health support (Reference: No)** |  |  |  |  |  |
| Yes | -6.446 (13.967) | -6.404 (13.605) | -5.688 (7.930) | -4.601 (6.935) | -4.537 (7.322) |
| **Legal advice services (Reference: No)** |  |  |  |  |  |
| Yes | 7.339 (12.035) | 7.351 (11.722) | 7.567 (6.832) | 7.893 (5.976) | 7.912 (6.309) |
| Age | 0.295 (11.391) | 0.310 (11.096) | 0.561 (6.467) | 0.942 (5.656) | 0.964 (5.971) |
| Gender | Omitted | Omitted | Omitted | Omitted | Omitted |
| Education | Omitted | Omitted | Omitted | Omitted | Omitted |
| Marital status (Reference: Married) | |  |  |  |  |
| Other | -3.660 (25.155) | -3.579 (24.502) | -2.204 (14.284) | -0.118 (12.491) | 0.003 (13.187) |
| Household per capita income | 1.612 (2.675) | 1.601 (2.606) | 1.413 (1.519) | 1.127 (1.328) | 1.110 (1.402) |
| Money transfers received from children | -2.358 (1.641) | -2.365 (1.598) | -2.479 (0.932) *** | -2.652 (0.815) ** | -2.662 (0.860) *** |
| Living arrangement (Reference: Living alone) | | | |  |  |
| Living with spouse | 33.779 (128.443) | 33.841 (124.435) | 34.904 (57.076) | 36.303 (42.215) | 36.426 (49.460) |
| Living with other family members | 6.618 (59.455) | 6.587 (57.600) | 6.056 (26.420) | 5.357 (19.541) | 5.295 (22.895) |
| Number of surviving children | -2.498 (4.307) | -2.510 (4.195) | -2.705 (2.445) | -3.000 (2.139) | -3.018 (2.258) |
| Having social health insurance (Reference: No) | |  |  |  |  |
| Yes | 6.094 (11.259) | 6.037 (10.967) | 5.071 (6.394) | 3.605 (5.591) | 3.519 (5.902) |
| Having old age pension (Reference: No) | |  |  |  |  |
| Yes | 10.456 (11.407) | 10.431 (11.111) | 9.998 (6.476) | 9.342 (5.664) | 9.304 (5.980) |
| Self-rated health (Reference: Bad) | |  |  |  |  |
| Fair | 3.399 (11.793) | 3.377 (11.487) | 3.000 (6.695) | 2.428 (5.856) | 2.395 (6.182) |
| Good | 1.829 (11.286) | 1.773 (10.993) | 0.830 (6.410) | -0.600 (5.604) | -0.684 (5.916) |
| Whether suffering from ﻿hypertension (Reference: No) |  |  |  |  |  |
| Yes | 6.676 (37.161) | 6.585 (36.001) | 5.046 (16.516) | 3.020 (12.213) | 2.842 (14.310) |
| Whether suffering from ﻿heart disease (Reference: No) |  |  |  |  |  |
| Yes | -2.232 (47.356) | -2.246 (45.878) | -2.489 (21.043) | -2.808 (15.564) | -2.836 (18.235) |
| Whether suffering from ﻿cardiovascular disease (Reference: No) |  |  |  |  |  |
| Yes | 2.655 (47.432) | 2.731 (45.952( | 4.031 (21.079) | 5.743 (15.589) | 5.893 (18.265) |
| Whether suffering from ﻿diabetes (Reference: No) |  |  |  |  |  |
| Yes | 2.655 (47.432) | 0.239 (92.872) | 3.408 (42.602) | 7.581 (31.507) | 7.947 (36.914) |
| Number of ADL limitations | 8.974 (1.643) *** | 8.979 (1.600) *** | 8.071 (0.933) *** | 8.210 (0.815) *** | 8.219 (0.861) *** |
| Cognitive function | -0.587 (0.570) | -0.583 (0.555) | -0.523 (0.323) | -0.431 (0.283) | -0.426 (0.298) |
| Year (Reference: 2005) |  |  |  |  |  |
| 2008 | -3.579 (38.890) | -3.556 (37.881) | -3.173 (22.078) | -2.591 (19.312) | -2.557 (20.387) |
| 2011 | -10.607 (72.431) | -10.612 (70.552) | -10.700 (41.119) | -10.834 (35.968) | -10.842 (37.971) |
| 2014 | -16.534 (101.250) | -16.609 (98.623) | -17.878 (57.479) | -19.803 (50.279) | -19.915 (53.079) |
| 2018 | -17.681 (147.328) | -17.610 (143.505) | -16.406 (83.638) | -14.581 (73.160) | -14.474 (77.234) |
| N | 6,307 | | | | |

Notes: ADL = activities of daily living. ﻿Cells represent ﻿coefficient (robust standard errors). *** p<0.01, ** p<0.05, * p<0.1.

**Supplementary Table 5. ﻿ Association between the availability of formal home and community-based care and total direct costs associated with informal care among rural residents: Quantile regression with FE models**

|  | **Direct costs associated with informal care** | | | | |
| --- | --- | --- | --- | --- | --- |
| **Variables** | **0.1** | **0.3** | **0.5** | **0.7** | **0.9** |
| **Daily living assistance (Reference: No)** |  |  |  |  |  |
| Yes | -0.083 (0.793) | -0.079 (0.766) | -0.023 (0.362) | 0.032 (0.362) | 0.035 (0.378) |
| **Community-based health care (Reference: No)** | |  |  |  |  |
| Yes | 0.057 (0.441) | 0.056 (0.426) | 0.031 (0.201) | 0.005 (0.201) | 0.004 (0.210) |
| **Mental health support (Reference: No)** |  |  |  |  |  |
| Yes | -0.675 (0.986) | -0.675 (0.950) | -0.684 (0.389) | -0.685 (0.411) | -0.685 (0.420) |
| **Legal advice services (Reference: No)** |  |  |  |  |  |
| Yes | 0.049 (0.715) | 0.049 (0.689) | 0.068 (0.249) | 0.081 (0.289) | 0.081 (0.304) |
| Age | 0.184 (0.407) | 0.184 (0.393) | 0.180 (0.185) | 0.177 (0.185) | 0.177 (0.194) |
| Gender | Omitted | Omitted | Omitted | Omitted | Omitted |
| Education | Omitted | Omitted | Omitted | Omitted | Omitted |
| Marital status (Reference: Married) | |  |  |  |  |
| Other | -1.617 (1.095) | -1.619 (1.058) | -1.648 (0.499) *** | -1.677 (0.499) *** | -1.678 (0.522) *** |
| Household per capita income | 0.085 (0.102) | 0.084 (0.099) | 0.062 (0.046) | 0.040 (0.046) | 0.039 (0.048) |
| Money transfers received from children | -0.040 (0.074) | -0.039 (0.071) | -0.032 (0.033) | -0.025 (0.033) | -0.025 (0.035) |
| Living arrangement (Reference: Living alone) | | | |  |  |
| Living with spouse | 0.156 (1.951) | 0.146 (1.877) | -0.022 (0.845) | -0.152 (0.939) | -0.162 (0.986) |
| Living with other family members | 0.292 (0.940) | 0.289 (0.905) | 0.248 (0.407) | 0.216 (0.452) | 0.213 (0.475) |
| Number of surviving children | -0.109 (0.191) | -0.109 (0.185) | -0.111 (0.087) | -0.112 (0.087) | -0.112 (0.091) |
| Having social health insurance (Reference: No) | |  |  |  |  |
| Yes | -0.264 (0.465) | -0.265 (0.449) | -0.273 (0.212) | -0.281 (0.212) | -0.281 (0.222) |
| Having old age pension (Reference: No) | |  |  |  |  |
| Yes | -0.579 (0.592) | -0.580 (0.572) | -0.602 (0.270) ** | -0.625 (0.270) ** | -0.626 (0.282) ** |
| Self-rated health (Reference: Bad) | |  |  |  |  |
| Fair | -0.682 (0.490) | -0.683 (0.473) | -0.694 (0.223) *** | -0.704 (0.223) *** | -0.705 (0.234) *** |
| Good | 0.054 (0.488) | 0.053 (0.471) | 0.026 (0.222) | 0.001 (0.222) | -0.001 (0.233) |
| Whether suffering from hypertension (Reference: No) |  |  |  |  |  |
| Yes | 0.631 (0.612) | 0.635 (0.588) | 0.717 (0.265) *** | 0.780 (0.294) *** | 0.785 (0.309) * |
| Whether suffering from heart disease (Reference: No) |  |  |  |  |  |
| Yes | 0.057 (0.775) | 0.054 (0.746) | 0.012 (0.336) | -0.020 (0.373) | -0.023 (0.392) |
| Whether suffering from cardiovascular disease (Reference: No) |  |  |  |  |  |
| Yes | -0.078 (0.769) | -0.075 (0.740) | -0.011 (0.333) | 0.037 (0.370) | 0.041 (0.389) |
| Whether suffering from diabetes (Reference: No) |  |  |  |  |  |
| Yes | -0.9211 (2.071) | -0.889 (1.993) | -0.367 (0.900) | 0.036 (0.997) | 0.066 (1.047) |
| Number of ADL limitations | 0.328 (0.121) *** | 0.327 (0.116) *** | 0.305 (0.055) *** | 0.283 (0.055) *** | 0.282 (0.057) *** |
| Cognitive function | 0.015 (0.024) | 0.015 (0.023) | 0.016 (0.011) | 0.016 (0.011) | 0.016 (0.011) |
| Year (Reference: 2005) |  |  |  |  |  |
| 2008 | 0.286 (1.400) | 0.288 (1.352) | 0.321 (0.638) | 0.354 (0.639) | 0.356 (0.668) |
| 2011 | 0.868 (2.598) | 0.874 (2.510) | 0.957 (1.185) | 1.040 (1.185) | 1.044 (1.240) |
| 2014 | -0.016 (3.642) | -0.010 (3.518) | 0.086 (1.661) | 0.183 (1.662) | 0.188 (1.738) |
| 2018 | 0.514 (5.232) | 0.513 (5.055) | 0.502 (2.386) | 0.491 (2.388) | 0.490 (2.498) |
| N | 6,307 | | | | |

Notes: Total direct costs include expenses related to transportation, medical supplies, and additional household needs. Cells represent ﻿coefficient (robust standard errors). *** p<0.01, ** p<0.05, * p<0.1.

**Supplementary Table 6. ﻿ Association between the availability of formal home and community-based care and hours of informal care among urban residents: Quantile regression with FE models**

|  | **Hours of informal care** | | | | |
| --- | --- | --- | --- | --- | --- |
| **Variables** | **0.1** | **0.3** | **0.5** | **0.7** | **0.9** |
| **Daily living assistance (Reference: No)** |  |  |  |  |  |
| Yes | -17.095 (17.859) | -17.371 (16.969) | -19.733 (10.054) ** | -23.217 (9.161) ** | -23.607 (10.062) ** |
| **Community-based health care (Reference: No)** | |  |  |  |  |
| Yes | 2.800 (12.591) | 2.870 (11.963) | 3.468 (7.081) | 4.349 (6.458) | 4.448 (7.093) |
| **Mental health support (Reference: No)** |  |  |  |  |  |
| Yes | -3.103 (11.928) | -3.142 (11.333) | -3.479 (6.708) | -3.977 (6.118) | -4.032 (6.720) |
| **Legal advice services (Reference: No)** |  |  |  |  |  |
| Yes | -6.179 (13.992) | -6.012 (13.294) | -4.578 (7.874) | -2.464 (7.177) | -2.227 (7.883) |
| Age | 6.695 (15.372) | 6.572 (14.606) | 5.519 (8.647) | 3.965 (7.885) | 3.791 (8.660) |
| Gender | Omitted | Omitted | Omitted | Omitted | Omitted |
| Education | Omitted | Omitted | Omitted | Omitted | Omitted |
| Marital status (Reference: Married) | |  |  |  |  |
| Other | 18.664 (22.712) | 18.890 (21.579) | 20.826 (12.777) | 23.682 (11.650) ** | 24.001 (12.795) * |
| Household per capita income | -1.724 (3.486) | -1.691 (3.312) | -1.408 (1.961) | -0.990 (1.788) | -0.943 (1.964) |
| Money transfers received from children | -1.348 (1.493) | -1.366 (1.419) | -1.521 (0.840) * | -1.749 (0.766) ** | -1.775 (0.841) ** |
| Living arrangement (Reference: Living alone) | | | |  |  |
| Living with spouse | 26.317 (41.986) | 26.394 (39.828) | 27.052 (22.985) | 27.990 (20.792) | 27.189 (28.488) |
| Living with other family members | -8.087 (24.262) | -8.072 (23.016) | -7.948 (13.282) | -7.770 (12.015) | -7.922 (13.803) |
| Number of surviving children | 3.468 (6.448) | 3.363 (0.191) | 2.458 (3.630) | 1.125 (3.307) | 0.976 (3.633) |
| Having social health insurance (Reference: No) | |  |  |  |  |
| Yes | 1.430 (12.118) | 1.413 (11.514) | 1.271 (6.814) | 1.062 (6.216) | 1.039 (6.827) |
| Having old age pension (Reference: No) | |  |  |  |  |
| Yes | 1.139 (15.378) | 0.874 (14.611) | -1.404 (8.659) | -4.764 (7.888) | -5.140 (8.664) |
| Self-rated health (Reference: Bad) | |  |  |  |  |
| Fair | -9.327 (12.768) | -9.540 (12.131) | --11.369 (7.190) | -14.065 (6.549) ** | -14.367 (7.193) ** |
| Good | -16.424 (13.339) | -16.529 (12.674) | -17.433 (7.503) ** | -18.765 (6.842) *** | -18.914 (7.515) ** |
| Whether suffering from hypertension (Reference: No) |  |  |  |  |  |
| Yes | 2.641 (13.469) | 2.744 (12.777) | 3.628 (7.376) | 4.890 (6.670) | 5.008 (7.233) |
| Whether suffering from heart disease (Reference: No) |  |  |  |  |  |
| Yes | -10.180 (14.583) | -10.419 (13.834) | -12.470 (7.995) | -15.399 (7.221) ** | -15.673 (7.832) ** |
| Whether suffering from cardiovascular disease (Reference: No) |  |  |  |  |  |
| Yes | 7.559 (15.766) | 7.732 (14.955) | 9.222 (8.636) | 11.348 (7.807) | 11.547 (8.467) |
| Whether suffering from diabetes (Reference: No) |  |  |  |  |  |
| Yes | -5.810 (29.325) | -5.896 (27.818) | -6.640 (16.055) | -7.702 (14.522) | -7.801 (15.749) |
| Number of ADL limitations | 8.089 (2.044) *** | 8.087 (1.943) *** | 8.066 (1.149) *** | 8.036 (1.048) *** | 8.032 (1.151) *** |
| Cognitive function | -0.280 (0.711) | -0.273 (0.676) | -0.216 (0.400) | -0.131 (0.365) | -0.121 (0.400) |
| Year (Reference: 2005) |  |  |  |  |  |
| 2008 | -4.408 (51.532) | -3.895 (48.961) | 0.500 (28.994) | 6.984 (26.433) | 7.710 (29.032) |
| 2011 | -25.847 (95.114) | -24.982 (90.370) | -17.551 (53.511) | -6.592 (48.789) | -5.366 (53.586) |
| 2014 | -35.377 (139.077) | -34.208 (132.141) | -24.175 (78.239) | -9.377 (71.340) | -7.721 (78.354) |
| 2018 | -8.040 (203.322) | -6.621 (193.180) | 5.563 (114.367) | 23.533 (104.294) | 25.544 (114.548) |
| N | 6,207 | | | | |

Notes: ADL = activities of daily living. ﻿Cells represent ﻿coefficient (robust standard errors). *** p<0.01, ** p<0.05, * p<0.1.

**Supplementary Table 7. ﻿ Association between the availability of formal home and community-based care and total direct costs associated with informal care among urban residents: Quantile regression with FE models**

|  | **Direct costs associated with informal care** | | | | |
| --- | --- | --- | --- | --- | --- |
| **Variables** | **0.1** | **0.3** | **0.5** | **0.7** | **0.9** |
| **Daily living assistance (Reference: No)** |  |  |  |  |  |
| Yes | 0.539 (1.106) | 0.527 (1.086) | 0.393 (0.984) | 0.274 (1.124) | 0.261 (1.151) |
| **Community-based health care (Reference: No)** | |  |  |  |  |
| Yes | -0.772 (1.221) | -0.772 (1.162) | -0.773 (0.461) * | -0.773 (0.372) ** | -0.773 (0.441) * |
| **Mental health support (Reference: No)** |  |  |  |  |  |
| Yes | -0.143 (0.602) | -0.143 (0.574) | -0.152 (0.334) | -0.161 (0.349) | -0.162 (0.366) |
| **Legal advice services (Reference: No)** |  |  |  |  |  |
| Yes | 0.016 (0.581) | 0.016 (0.554) | 0.014 (0.323) | 0.012 (0.336) | 0.012 (0.354) |
| Age | 0.283 (1.913) | 0.285 (1.821) | 0.299 (0.723) | 0.314 (0.583) | 0.316 (0.692) |
| Gender | Omitted | Omitted | Omitted | Omitted | Omitted |
| Education | Omitted | Omitted | Omitted | Omitted | Omitted |
| Marital status (Reference: Married) | |  |  |  |  |
| Other | 1.016 (3.037) | 0.993 (2.890) | 0.723 (1.148) | 0.431 (0.926) | 0.404 (1.098) |
| Household per capita income | -0.202 (0.432) | -0.202 (0.411) | -0.195 (0.163) | -0.187 (0.131) | -0.187 (0.156) |
| Money transfers received from children | 0.021 (0.185) | 0.020 (0.176) | 0.016 (0.070) | 0.011 (0.056) | 0.011 (0.067) |
| Living arrangement (Reference: Living alone) | | | |  |  |
| Living with spouse | 0.843 (3.131) | 0.865 (2.920) | 1.042 (0.172) | 1.240 (1.273) | 1.262 (1.436) |
| Living with other family members | 1.435 (1.463) | 1.443 (1.365) | 0.315 (0.088) ** | 1.563 (0.595) *** | 1.570 (0.671) ** |
| Number of surviving children | -0.030 (0.791) | -0.026 (0.753) | 0.020 (0.299) | 0.070 (0.241) | 0.075 (0.286) |
| Having social health insurance (Reference: No) | |  |  |  |  |
| Yes | -0.202 (1.379) | -0.192 (1.313) | -0.069 (0.521) | 0.062 (0.420) | 0.075 (0.498) |
| Having old age pension (Reference: No) | |  |  |  |  |
| Yes | 0.187 (1.702) | 0.180 (1.620) | 0.101 (0.643) | 0.015 (0.519) | 0.007 (0.615) |
| Self-rated health (Reference: Bad) | |  |  |  |  |
| Fair | -0.570 (1.543) | -0.571 (1.468) | -0.572 (0.583) | -0.574 (0.470) | -0.574 (0.558) |
| Good | -0.989 (1.522) | -0.987 (1.448) | -0.959 (0.575) * | -0.930 (0.464) | -0.927 (0.550) * |
| Whether suffering from ﻿hypertension (Reference: No) |  |  |  |  |  |
| Yes | 0.419 (0.809) | 0.426 (0.755) | 0.482 (0.357) | 0.545 (0.329) | 0.552 (0.371) |
| Whether suffering from﻿ heart disease (Reference: No) |  |  |  |  |  |
| Yes | -0.458 (0.903) | -0.464 (0.842) | -0.512 (0.398) | -0.565 (0.367) | -0.571 (0.414) |
| Whether suffering from ﻿cardiovascular (Reference: No) |  |  |  |  |  |
| Yes | -0.016 (0.956) | -0.007 (0.892) | 0.062 (0.421) | 0.141 (0.389) | 0.150 (0.438) |
| Whether suffering from ﻿diabetes (Reference: No) |  |  |  |  |  |
| Yes | 0.501 (1.865) | 0.470 (1.740) | 0.222 (0.823) | -0.053 (0.759) | -0.084 (0.855) |
| Number of ADL limitations | 0.295 (0.371) | 0.294 (0.353) | 0.281 (0.140) ** | 0.267 (0.113) | 0.265 (0.134) ** |
| Cognitive function | 0.014 (0.080) | 0.014 (0.076) | 0.014 (0.030) | 0.014 (0.024) | 0.014 (0.029) |
| Year (Reference: 2005) |  |  |  |  |  |
| 2008 | 0.711 (6.234) | 0.711 (5.932) | 0.708 (2.355) | 0.706 (1.901) | 0.706 (2.254) |
| 2011 | 0.927 (11.806) | 0.923 (11.235) | 0.876 (4.460) | 0.825 (3.600) | 0.820 (4.270) |
| 2014 | 0.273 (17.265) | 0.266 (16.430) | 0.181 (6.523) | 0.090 (5.264) | 0.081 (6.244) |
| 2018 | -1.421 (24.948) | -1.425 (23.742) | -1.482 (9.426) | -1.542 (7.607) | -1.548 (9.023) |
| N | 6,207 | | | | |

Notes: Total direct costs include expenses related to transportation, medical supplies, and additional household needs. Cells represent ﻿coefficient (robust standard errors). *** p<0.01, ** p<0.05, * p<0.1.

**Supplementary Table 8. Association between the availability of formal home and community-based care and hours of informal care (Ln)**

| **Variables** | **Model 1** | **Model 2** | **Model 3** | **Model 4** | **Model 5** |
| --- | --- | --- | --- | --- | --- |
| **Daily living assistance (Reference: No)** | | |  |  |  |
| Yes | -0.264 (0.130) ** | |  |  | -0.257 (0.132) ** |
| **Community-based health care (Reference: No)** | | | |  |  |
| Yes |  | 0.024 (0.081) |  |  | 0.047 (0.082) |
| **Mental health support (Reference: No)** | | |  |  |  |
| Yes |  |  | -0.150 (0.094) |  | -0.109 (0.105) |
| **Legal advice services (Reference: No)** | | | |  |  |
| Yes |  |  |  | 0.002 (0.084) | 0.054 (0.094) |
| Age | 0.081 (0.072) | 0.085 (0.072) | 0.082 (0.072) | 0.089 (0.074) | 0.086 (0.073) |
| Gender | Omitted | Omitted | Omitted | Omitted | Omitted |
| Education | Omitted | Omitted | Omitted | Omitted | Omitted |
| Marital status (Reference: Married) | | |  |  |  |
| Other | 0.191 (0.192) | 0.188 (0.192) | 0.216 (0.194) | 0.185 (0.195) | 0.187 (0.192) |
| Household per capita income | -0.001 (0.026) | 0.001 (0.026) | -0.000 (0.026) | 0.001 (0.026) | -0.001 (0.026) |
| Money transfers received from children | -0.017 (0.012) | -0.018 (0.011) | -0.016 (0.011) | -0.015 (0.011) | -0.018 (0.011) |
| Residence (Reference: Rural) | | |  |  |  |
| Urban | -0.067 (0.093) | -0.058 (0.093) | -0.052 (0.093) | -0.054 (0.093) | -0.065 (0.093) |
| Living arrangement (Reference: Living alone) | | | | |  |
| Living with spouse | 0.203 (0.350) | 0.179 (0.349) | 0.314 (0.365) | 0.201 (0.353) | 0.345 (0.366) |
| Living with other family members | -0.091 (0.177) | -0.105 (0.176) | -0.092 (0.176) | -0.092 (0.177) | -0.096 (0.178) |
| Number of surviving children | -0.050 (0.042) | -0.046 (0.043) | -0.045 (0.042) | -0.045 (0.042) | -0.048 (0.042) |
| Having social health insurance (Reference: No) | | |  |  |  |
| Yes | -0.151 (0.090) * | -0.143 (0.090) | -0.148 (0.091) | -0.135 (0.092) | -0.153 (0.091) |
| Having old age pension (Reference: No) | | |  |  |  |
| Yes | 0.120 (0.027) | 0.081 (0.105) | 0.089 (0.105) | 0.092 (0.106) | 0.117 (0.106) |
| Self-rated health (Reference: Bad) | | |  |  |  |
| Fair | 0.079 (0.092) | 0.058 (0.091) | 0.064 (0.091) | 0.066 (0.092) | 0.071 (0.092) |
| Good | 0.058 (0.096) | 0.042 (0.096) | 0.043 (0.096) | 0.038 (0.097) | 0.053 (0.097) |
| Whether suffering from ﻿hypertension (Reference: No) |  |  |  |  |  |
| Yes | 0.150 (0.103) | 0.159 (0.103) | 0.173 (0.104) | 0.167 (0.104) | 0.146 (0.104) |
| Whether suffering from ﻿heart disease (Reference: No) |  |  |  |  |  |
| Yes | -0.119 (0.124) | -0.103 (0.124) | -0.110 (0.124) | -0.126 (0.125) | -0.139 (0.125) |
| Whether suffering from ﻿cardiovascular disease (Reference: No) |  |  |  |  |  |
| Yes | -0.110 (0.126) | -0.121 (0.127) | -0.108 (0.127) | -0.098 (0.128) | -0.122 (0.129) |
| Whether suffering from ﻿diabetes (Reference: No) |  |  |  |  |  |
| Yes | 0.022 (0.270) | 0.008 (0.267) | 0.002 (0.268) | -0.029 (0.271) | -0.004 (0.274) |
| Number of ADL limitations | 0.248 (0.024) *** | 0.243 (0.024) *** | 0.244 (0.024) *** | 0.243 (0.024) *** | 0.246 (0.024) *** |
| Cognitive function | -0.011 (0.005) ** | -0.012 (0.005) *** | -0.011 (0.004) ** | -0.012 (0.004) *** | -0.011 (0.005) ** |
| Year (Reference: 2005) | |  |  |  |  |
| 2008 | -0.068 (0.254) | -0.103 (0.254) | -0.085 (0.254) | -0.111 (0.261) | -0.077 (0.255) |
| 2011 | -0.379 (0.466) | -0.423 (0.465) | -0.381 (0.466) | -0.449 (0.479) | -0.417 (0.467) |
| 2014 | -0.508 (0.659) | -0.573 (0.658) | -0.517 (0.659) | -0.613 (0.677) | -0.555 (0.661) |
| 2018 | -0.197 (0.960) | -0.251 (0.959) | -0.204 (0.960) | -0.328 (00.984) | -0.237 (0.962) |
| N | 12,514 | | | | |

Notes: ADL = activities of daily living. ﻿Cells represent ﻿coefficient (robust standard errors). *** p<0.01, ** p<0.05, * p<0.1.

**Supplementary Table 9. ﻿ Association between the availability of formal home and community-based care and hours of informal care (Ln): Quantile regression with FE models**

|  | **Hours of informal care** | | | | |
| --- | --- | --- | --- | --- | --- |
| **Variables** | **0.1** | **0.3** | **0.5** | **0.7** | **0.9** |
| **Daily living assistance (Reference: No)** |  |  |  |  |  |
| Yes | -0.231 (0.356) | -0.233 (0.341) | -0.263 (0.140) ** | -0.292 (0.127) ** | -0.294 (0.137) ** |
| **Community-based health care (Reference: No)** | |  |  |  |  |
| Yes | 0.040 (0.239) | 0.040 (0.228) | 0.055 (0.093) | 0.068 (0.084) | 0.070 (0.092) |
| **Mental health support (Reference: No)** |  |  |  |  |  |
| Yes | -0.129 (0.180) | -0.129 (0.174) | -0.120 (0.097) | -0.111 (0.106) | -0.110 (0.111) |
| **Legal advice services (Reference: No)** |  |  |  |  |  |
| Yes | 0.105 (0.110) | 0.037 (0.171) | 0.052 (0.095) | 0.067 (0.104) | 0.069 (0.109) |
| Age | 0.093 (0.164) | 0.092 (0.156) | 0.080 (0.064) | 0.067 (0.058) | 0.067 (0.063) |
| Gender | Omitted | Omitted | Omitted | Omitted | Omitted |
| Education | Omitted | Omitted | Omitted | Omitted | Omitted |
| Marital status (Reference: Married) | |  |  |  |  |
| Other | 0.182 (0.575) | 0.183 (0.551) | 0.195 (0.225) | 0.205 (0.204) | 0.207 (0.222) |
| Household per capita income | 0.001 (0.072) | 0.001 (0.069) | 0.001 (0.028) | 0.001 (0.025) | 0.001 (0.028) |
| Money transfers received from children | -0.014 (0.034) | -0.014 (0.032) | -0.020 (0.013) | -0.025 (0.012) ** | -0.025 (0.013) ** |
| Residence (Reference: Rural) |  |  |  |  |  |
| Urban | -0.100 (0.272) | -0.097 (0.260) | -0.070 (0.106) | -0.043 (0.096) | -0.041 (0.105) |
| Living arrangement (Reference: Living alone) | | | |  |  |
| Living with spouse | 0.411 (6.014) | 0.406 (5.719) | 0.349 (2.335) | 0.284 (11.674) | 0.279 (1.958) |
| Living with other family members | -0.086 (3.430) | -0.087 (3.262) | -0.095 (1.331) | -0.104 (0.995) | -0.105 (1.116) |
| Number of surviving children | -0.063 (0.120) | -0.063 (0.115) | -0.058 (0.047) | -0.053 (0.043) | -0.053 (0.046) |
| Having social health insurance (Reference: No) | |  |  |  |  |
| Yes | -0.156 (0.245) | -0.157 (0.235) | -0.166 (0.096) * | -0.175 (0.087) ** | -0.176 (0.094) * |
| Having old age pension (Reference: No) | |  |  |  |  |
| Yes | 0.101 (0.296) | 0.101 (0.284) | 0.103 (0.116) | 0.105 (0.104) | 0.105 (0.114) |
| Self-rated health (Reference: Bad) | |  |  |  |  |
| Fair | 0.117 (0.264) | 0.116 (0.253) | 0.102 (0.103) | 0.089 (0.093) | 0.088 (0.101) |
| Good | 0.069 (0.266) | 0.067 (0.255) | 0.057 (0.104) | 0.045 (0.094) | 0.044 (0.103) |
| Whether suffering from﻿ hypertension (Reference: No) |  |  |  |  |  |
| Yes | 0.169 (1.883) | 0.167 (1.791) | 0.147 (0.731) | 0.125 (0.524) | 0.124 (0.613) |
| Whether suffering from ﻿heart disease (Reference: No) |  |  |  |  |  |
| Yes | -0.098 (2.371) | -0.101 (2.255) | -0.137 (0.920) | -0.177 (0.660) | -0.181 (0.772) |
| Whether suffering from ﻿cardiovascular disease (Reference: No) |  |  |  |  |  |
| Yes | -0.198 (2.230) | -0.192 (2.120) | -0.126 (0.865) | -0.0511 (0.620) | -0.045 (0.726) |
| Whether suffering from ﻿diabetes (Reference: No) |  |  |  |  |  |
| Yes | -0.022 (4.968) | -0.020 (4.724) | -0.005 (1.928) | 0.012 (1.382) | 0.013 (1.617) |
| Number of ADL limitations | 0.141 (0.038) *** | 0.140 (0.037) *** | 0.139 (0.015) *** | 0.138 (0.013) *** | 0.137 z90.015) *** |
| Cognitive function | -0.012 (0.013) | -0.012 (0.013) | -0.010 (0.005) ** | -0.009 (0.004) * | -0.009 (0.005) * |
| Year (Reference: 2005) |  |  |  |  |  |
| 2008 | -0.080 (0.577) | -0.078 (0.554) | -0.047 (0.226) | -0.017 (0.205) | -0.014 (0.223) |
| 2011 | -0.459 (1.052) | -0.455 (1.009) | -0.386 (0.411) | -0.320 (0.373) | -0.315 (0.406) |
| 2014 | -0.631 (1.477) | -0.624 (1.415) | -0.511 (0.577) | -0.410 (0.523) | -0.393 (0.570) |
| 2018 | -0.273 (2.151) | -0.265 (2.062) | -0.138 (0.841) | -0.014 (0.763) | -0.006 (0.831) |
| N | 12,514 | | | | |

Notes: ADL = activities of daily living. ﻿Cells represent ﻿coefficient (robust standard errors). *** p<0.01, ** p<0.05, * p<0.1.

**Supplementary Table 10. Subgroup analysis in the association between availability of formal home and community-based care and hours of informal care (Ln)**

| **Variables** | **0.1** | **0.3** | **0.5** | **0.7** | **0.9** | **Total rural/urban sample** |
| --- | --- | --- | --- | --- | --- | --- |
| **Rural** |  |  |  |  |  |  |
| **Daily living assistance (Reference: No)** | | |  |  |  |  |
| Yes | -0.226 (0.480) | -0.228 (0.463) | -0.257 (0.201) | -0.287 (0.189) | -0.289 (0.204) | -0.236 (0.226) |
| **Community-based health care (Reference: No)** | | |  |  |  |  |
| Yes | 0.167 (0.327) | 0.166 (0.315) | 0.151 (0.137) | 0.135 (0.128) | 0.135 (0.138) | 0.126 (0.149) |
| **Mental health support (Reference: No)** | | | | | | |
| Yes | -0.264 (0.410) | -0.263 (0.398) | -0.239 (0.197) | -0.209 (0.163) | -0.207 (0.175) | -0.236 (0.206) |
| **Legal advice services (Reference: No)** | | | | | | |
| Yes | 0.115 (0.346) | 0.116 (0.336) | 0.130 (0.166) | 0.148 (0.138) | 0.149 (0.148) | 0.132 (0.160) |
| **Urban** |  |  |  |  |  |  |
| **Daily care (Reference: No)** | | |  |  |  |  |
| Yes | -0.407 (0.475) | -0.406 (0.455) | -0.402 (0.253) | -0.396 (0.176) ** | -0.395 (0.195) ** | -0.401 (0.203) ** |
| **Medical care (Reference: No)** | | |  |  |  |  |
| Yes | -0.037 (0.245) | -0.036 (0.237) | -0.022 (0.184) | 0.001 (0.198) | 0.003 (0.206) | -0.017 (0.121) |
| **Mental health support (Reference: No)** | | | | | | |
| Yes | -0.101 (0.222) | -0.101 (0.212) | -0.107 (0.119) | -0.115 (0.110) | -0.116 (0.120) | -0.108 (0.148) |
| **Legal advice services (Reference: No)** | | | | | | |
| Yes | -0.121 (0.261) | -0.120 (0.249) | -0.104 (0.139) | -0.082 (0.129) | -0.080 (0.141) | -0.101 (0.151) |

Notes: Total rural sample is 6,307. Total urban sample is 6,207. All the models control for needs-related variables and socioeconomic-related variables. Cells represent ﻿coefficient (robust standard errors). *** p<0.01, ** p<0.05, * p<0.1.

**Supplementary Table 11. ﻿Association between the availability of formal home and community-based care and informal care: FE models**

| **Variables** | **Hours of informal care (Ln)** | **Total direct costs associated with informal care** |
| --- | --- | --- |
| **Daily living assistance (Reference: No)** | | |
| Yes | -0.257 (0.132) ** | -0.192 (0.268) |
| **Community-based health care (Reference: No)** | | |
| Yes | 0.048 (0.082) | -0.320 (0.153) ** |
| **Mental health support (Reference: No)** | | |
| Yes | -0.109 (0.105) | -0.383 (0.215) * |
| **Legal advice services (Reference: No)** | | |
| Yes | 0.055 (0.094) | 0.053 (0.189) |
| GDP per capita | -0.162 (0.522) | -1.155 (1.058) |
| Age | 0.086 (0.073) | 0.299 (0.143) ** |
| Gender | Omitted | Omitted |
| Education | Omitted | Omitted |
| Marital status (Reference: Married) | | |
| Other | 0.185 (0.192) | -0.386 (0.414) |
| Household per capita income | -0.001 (0.026) | -0.009 (0.057) |
| Money transfers received from children | -0.018 (0.011) | 0.023 (0.025) |
| Residence (Reference: Rural) | | |
| Urban | -0.064 (0.093) | 0.001 (0.190) |
| Living arrangement (Reference: Living alone) | | |
| Living with spouse | 0.345 (0.367) | 0.773 (0.754) |
| Living with other family members | -0.068 (0.179) | 0.532 (0.382) |
| Number of surviving children | -0.048 (0.042) | -0.058 (0.084) |
| Having social health insurance (Reference: No) | | |
| Yes | -0.150 (0.091) | -0.174 (0.183) |
| Having old age pension (Reference: No) | | |
| Yes | 0.117 (0.106) | 0.037 (0.221) |
| Self-rated health (Reference: Bad) | | |
| Fair | 0.071 (0.092) | -0.334 (0.190Z) * |
| Good | 0.053 (0.097) | -0.233 (0.195) |
| Whether suffering from diabetes (Reference: No) |  |  |
| Yes | 0.150 (0.105) | 0.385 (0.218) * |
| Whether suffering from diabetes (Reference: No) |  |  |
| Yes | -0.138 (0.126) | -0.371 (0.262) |
| Whether suffering from diabetes (Reference: No) |  |  |
| Yes | -0.163 (0.130) | 0.044 (0.270) |
| Whether suffering from diabetes (Reference: No) |  |  |
| Yes | -0.013 (0.275) | 0.263 (0.536) |
| Number of ADL limitations | 0.246 (0.024) *** | 0.315 (0.048) *** |
| Cognitive function | -0.011 (0.005) ** | 0.010 (0.010) |
| Year (Reference: 2005) | | |
| 2008 | -0.077 (0.255) | 0.199 (0.502) |
| 2011 | -0.417 (0.467) | 0.264 (0.920) |
| 2014 | -0.555 (0.661) | -0.615 (1.296) |
| 2018 | -0.237 (0.962) | -1.479 (1.881) |
| N | 12,514 | |

Notes: Total direct costs include expenses related to transportation, medical supplies, and additional household needs. Cells represent ﻿coefficient (robust standard errors). *** p<0.01, ** p<0.05, * p<0.1.

**Supplementary Table 12. Association between the availability of formal home and community-based care and informal care: Quantile regression with FE models (taking GDP per capita into model)**

|  | **Hours of informal care (Ln)** | | | | |
| --- | --- | --- | --- | --- | --- |
| **Variables** | **0.1** | **0.3** | **0.5** | **0.7** | **0.9** |
| **Daily living assistance (Reference: No)** | |  |  |  |  |
| Yes | -0.231 (0.221) | -0.233 (0.214) | -0.264 (0.129) ** | -0.292 (0.146) ** | -0.294 (0.137) ** |
| **Community-based health care (Reference: No)** | |  |  |  |  |
| Yes | 0.042 (0.148) | 0.043 (0.143) | 0.058 (0.086) | 0.070 (0.098) | 0.069 (0.092) |
| **Mental health support (Reference: No)** |  |  |  |  |  |
| Yes | -0.259 (1.987) | -0.258 (2.004) | -0.235 (2.300) | -0.206 (2.722) | -0.205 (2.750) |
| **Legal advice services (Reference: No)** |  |  |  |  |  |
| Yes | 0.109 (1.674) | 0.109 (1.688) | 0.123 (1.938) | 0.140 (2.293) | 0.1411 (2.316) |
|  | **Total direct costs associated with informal care** | | | | |
| **Variables** | **0.1** | **0.3** | **0.5** | **0.7** | **0.9** |
| **Daily living assistance (Reference: No)** | |  |  |  |  |
| Yes | -0.187 (0.515) | -0.187 (0.495) | -0.191 (0.223) | -0.196 (0.223) | -0.196 (0.236) |
| **Community-based health care (Reference: No)** | |  |  |  |  |
| Yes | -0.272 (0.325) | -0.274 (0.312) | -0.311 (0.141) ** | -0.345 (0.141) ** | -0.348 (0.149) ** |
| **Mental health support (Reference: No)** |  |  |  |  |  |
| Yes | -0.100 (0.232) | -0.101 (0.221) | -0.106 (0.128) | -0.115 (0.109) | -0.116 (0.119) |
| **Legal advice services (Reference: No)** |  |  |  |  |  |
| Yes | -0.120 (0.273) | -0.119 (0.260) | -0.104 (0.150) | -0.081 (0.128) | -0.079 (0.141) |
| **N** | 12,514 | | | | |

Notes: Total direct costs include expenses related to transportation, medical supplies, and additional household needs. All the models control for needs-related variables and socioeconomic-related variables. *** p<0.01, ** p<0.05, * p<0.1.

**Supplementary Table 13. Subgroup analysis in the association between availability of formal home and community-based care and informal care (taking GDP per capita into model)**

|  | **Hours of informal care (Ln)** | | | | |  |
| --- | --- | --- | --- | --- | --- | --- |
| **Variables** | **0.1** | **0.3** | **0.5** | **0.7** | **0.9** | **Total rural/urban sample** |
| **Rural** |  |  |  |  |  |  |
| **Daily living assistance (Reference: No)** | | |  |  |  |  |
| **Yes** | -0.224 (0.376) | -0.226 (0.364) | -0.265 (0.159) | -0.297 (0.175) | -0.299 (0.185) | -0.262 (0.226) |
| **Community-based health care (Reference: No)** | | |  |  |  |  |
| **Yes** | 0.138 (0.256) | 0.137 (0.247) | 0.119 (0.108) | 0.105 (0.120) | 0.104 (0.126) | 0.122 (0.149) |
| **Mental health support (Reference: No)** | | | | | | |
| **Yes** | -6.446 (13.967) | -6.404 (13.605) | -5.688 (7.930) | -4.601 (6.935) | -4.537 (7.322) | -5.506 (9.647) |
| **Legal advice services (Reference: No)** | | | | | | |
| **Yes** | 7.339 (12.035) | 7.351 (11.722) | 7.567 (6.832) | 7.893 (5.976) | 7.912 (6.309) | 7.621 (7.510) |
| **Urban** |  |  |  |  |  |  |
| **Daily living assistance (Reference: No)** | | |  |  |  |  |
| **Yes** | -0.409 (0.475) | -0.408 (0.455) | -0.406 (0.253) | -0.402 (0.186) ** | -0.402 (0.197) ** | -0.405 (0.203) ** |
| **Community-based health care (Reference: No)** | | |  |  |  |  |
| **Yes** | -0.037 (0.245) | -0.036 (0.237) | -0.022 (0.184) | 0.001 (0.198) | 0.003 (0.206) | -0.013 (0.121) |
| **Mental health support (Reference: No)** | | | | | | |
| **Yes** | -3.103 (11.928) | -3.142 (11.333) | -3.479 (6.708) | -3.977 (6.118) | -4.032 (6.720) | -3.557 (7.073) |
| **Legal advice services (Reference: No)** | | | | | | |
| **Yes** | -6.179 (13.992) | -6.012 (13.294) | -4.578 (7.874) | -2.464 (7.177) | -2.227 (7.883) | -4.251 (7.213) |
|  | **Total direct costs associated with informal care** | | | | |  |
| **Variables** | **0.1** | **0.3** | **0.5** | **0.7** | **0.9** | **Total rural/urban sample** |
| **Rural** |  |  |  |  |  |  |
| **Daily living assistance (Reference: No)** | | |  |  |  |  |
| **Yes** | -0.073 (0.809) | -0.069 (0.780) | 0.004 (0.316) | 0.055 (0.369) | 0.058 (0.387) | -0.007 (0.441) |
| **Community-based health care (Reference: No)** | | |  |  |  |  |
| **Yes** | 0.057 (0.448) | 0.056 (0.432) | 0.031 (0.175) | 0.014 (0.204) | 0.013 (0.214) | 0.035 (0.291) |
| **Mental health support (Reference: No)** | | | | | | |
| **Yes** | -0.675 (0.986) | -0.675 (0.950) | -0.684 (0.389) | -0.685 (0.411) | -0.685 (0.420) | -0.680 (0.401) |
| **Legal advice services (Reference: No)** | | | | | | |
| **Yes** | 0.049 (0.715) | 0.049 (0.689) | 0.068 (0.249) | 0.081 (0.289) | 0.081 (0.304) | 0.065 (0.302) |
| **Urban** |  |  |  |  |  |  |
| **Daily living assistance (Reference: No)** | | |  |  |  |  |
| **Yes** | 0.539 (1.106) | 0.527 (1.086) | 0.393 (0.984) | 0.274 (1.124) | 0.261 (1.151) | 0.401 (0.449) |
| **Community-based health care (Reference: No)** | | |  |  |  |  |
| **Yes** | -0.772 (1.221) | -0.772 (1.162) | -0.773 (0.461) * | -0.773 (0.372) ** | -0.773 (0.441) * | -0.773 (0.262) *** |
| **Mental health support (Reference: No)** | | | | | | |
| **Yes** | -0.143 (0.602) | -0.143 (0.574) | -0.152 (0.334) | -0.161 (0.349) | -0.162 (0.366) | -0.152 (0.318) |
| **Legal advice services (Reference: No)** | | | | | | |
| **Yes** | 0.016 (0.581) | 0.016 (0.554) | 0.014 (0.323) | 0.012 (0.336) | 0.012 (0.354) | 0.014 (0.340) |

Notes: Total rural sample is 6,307. Total urban sample is 6,207. Total direct costs include expenses related to transportation, medical supplies, and additional household needs. All the models control for needs-related variables and socioeconomic-related variables. Cells represent ﻿coefficient (robust standard errors). *** p<0.01, ** p<0.05, * p<0.1.

**Supplementary Table 14. ﻿Association between the availability of formal home and community-based care and informal care: FE models (taking instrumental activities of daily living and health care utilization into models)**

| **Variables** | **Hours of informal care** | **Total direct costs associated with informal care** |
| --- | --- | --- |
| **Daily living assistance (Reference: No)** | | |
| Yes | -0.253 (0.132) ** | -0.188 (0.268) |
| **Community-based health care (Reference: No)** | | |
| Yes | 0.045 (0.082) | -0.328 (0.169) ** |
| **Mental health support (Reference: No)** | | |
| Yes | -0.100 (0.105) | -0.310 (0.213) |
| **Legal advice services (Reference: No)** | | |
| Yes | 0.049 (0.094) | 0.004 (0.188) |
| GDP per capita | -0.092 (0.529) | -1.206 (1.061) |
| Number of limitations in instrumental activities of daily living | 0.038 (0.024) | 0.047 (0.053) |
| Use health care (Reference: No) | | |
| Yes | 0.100 (0.102) | 0.988 (0.205) *** |
| Age | 0.077 (0.073) | 0.281 (0.143) ** |
| Gender | Omitted | Omitted |
| Education | Omitted | Omitted |
| Marital status (Reference: Married) | | |
| Other | 0.185 (0.192) | -0.386 (0.414) |
| Household per capita income | -0.001 (0.026) | -0.009 (0.057) |
| Money transfers received from children | -0.018 (0.011) | 0.023 (0.025) |
| Residence (Reference: Rural) | | |
| Urban | -0.064 (0.093) | 0.001 (0.190) |
| Living arrangement (Reference: Living alone) | | |
| Living with spouse | 0.328 (0.367) | 0.722 (0.754) |
| Living with other family members | -0.088 (0.179) | 0.483 (0.383) |
| Number of surviving children | -0.048 (0.042) | -0.058 (0.084) |
| Having social health insurance (Reference: No) | | |
| Yes | -0.150 (0.091) | -0.174 (0.183) |
| Having old age pension (Reference: No) | | |
| Yes | 0.117 (0.106) | 0.037 (0.221) |
| Self-rated health (Reference: Bad) | | |
| Fair | 0.071 (0.092) | -0.334 (0.190Z) * |
| Good | 0.053 (0.097) | -0.233 (0.195) |
| Whether suffering from hypertension (Reference: No) |  |  |
| Yes | 0.142 (0.105) | 0.374 (0.218) * |
| Whether suffering from heart disease (Reference: No) |  |  |
| Yes | -0.138 (0.126) | -0.367 (0.262) |
| Whether suffering from cardiovascular disease (Reference: No) |  |  |
| Yes | -0.158 (0.130) | 0.053 (0.270) |
| Whether suffering from diabetes (Reference: No) |  |  |
| Yes | 0.004 (0.275) | 0.308 (0.536) |
| Number of ADL limitations | 0.246 (0.024) *** | 0.315 (0.048) *** |
| Cognitive function | -0.011 (0.005) ** | 0.010 (0.010) |
| Year (Reference: 2005) | | |
| 2008 | -0.077 (0.255) | 0.199 (0.502) |
| 2011 | -0.417 (0.467) | 0.264 (0.920) |
| 2014 | -0.555 (0.661) | -0.615 (1.296) |
| 2018 | -0.237 (0.962) | -1.479 (1.881) |
| N | 12,514 | |

Notes: Total direct costs include expenses related to transportation, medical supplies, and additional household needs. Cells represent ﻿coefficient (robust standard errors). *** p<0.01, ** p<0.05, * p<0.1.

**Supplementary Table 15. Association between the availability of formal home and community-based care and informal care: Quantile regression with FE models (taking instrumental activities of daily living and health care utilization into models)**

|  | **Hours of informal care (Ln)** | | | | |
| --- | --- | --- | --- | --- | --- |
| **Variables** | **0.1** | **0.3** | **0.5** | **0.7** | **0.9** |
| **Daily living assistance (Reference: No)** | |  |  |  |  |
| Yes | -0.231 (0.221) | -0.233 (0.214) | -0.264 (0.129) ** | -0.292 (0.146) ** | -0.294 (0.137) ** |
| **Community-based health care (Reference: No)** | |  |  |  |  |
| Yes | 0.038 (0.163) | 0.039 (0.156) | 0.054 (0.078) | 0.066 (0.085) | 0.067 (0.089) |
| **Mental health support (Reference: No)** |  |  |  |  |  |
| Yes | -0.259 (1.987) | -0.258 (2.004) | -0.235 (2.300) | -0.206 (2.722) | -0.205 (2.750) |
| **Legal advice services (Reference: No)** |  |  |  |  |  |
| Yes | 0.109 (1.674) | 0.109 (1.688) | 0.123 (1.938) | 0.140 (2.293) | 0.1411 (2.316) |
|  | **Total direct costs associated with informal care** | | | | |
| **Variables** | **0.1** | **0.3** | **0.5** | **0.7** | **0.9** |
| **Daily living assistance (Reference: No)** | |  |  |  |  |
| Yes | -0.179 (0.526) | -0.179 (0.507) | -0.189 (0.203) | -0.196 (0.235) | -0.197 (0.247) |
| **Community-based health care (Reference: No)** | |  |  |  |  |
| Yes | -0.279 (0.331) | -0.282 (0.318) | -0.337 (0.128) *** | -0.375 (0.148) *** | -0.377 (0.155) ** |
| **Mental health support (Reference: No)** |  |  |  |  |  |
| Yes | -0.100 (0.232) | -0.101 (0.221) | -0.106 (0.128) | -0.115 (0.109) | -0.116 (0.119) |
| **Legal advice services (Reference: No)** |  |  |  |  |  |
| Yes | -0.120 (0.273) | -0.119 (0.260) | -0.104 (0.150) | -0.081 (0.128) | -0.079 (0.141) |
| **N** | 12,514 | | | | |

Notes: Total direct costs include expenses related to transportation, medical supplies, and additional household needs. All the models control for needs-related variables and socioeconomic-related variables. *** p<0.01, ** p<0.05, * p<0.1.

**Supplementary Table 16. Subgroup analysis in the association between availability of formal home and community-based care and informal care (taking instrumental activities of daily living into model and health care utilization into models)**

|  | **Hours of informal care (Ln)** | | | | |  |
| --- | --- | --- | --- | --- | --- | --- |
| **Variables** | **0.1** | **0.3** | **0.5** | **0.7** | **0.9** | **Total rural/urban sample** |
| **Rural** |  |  |  |  |  |  |
| **Daily living assistance (Reference: No)** | | |  |  |  |  |
| **Yes** | -0.224 (0.376) | -0.226 (0.364) | -0.265 (0.159) | -0.297 (0.175) | -0.299 (0.185) | -0.262 (0.226) |
| **Community-based health care (Reference: No)** | | |  |  |  |  |
| **Yes** | 0.138 (0.256) | 0.137 (0.247) | 0.119 (0.108) | 0.105 (0.120) | 0.104 (0.126) | 0.122 (0.149) |
| **Mental health support (Reference: No)** | | | | | | |
| **Yes** | -6.446 (13.967) | -6.404 (13.605) | -5.688 (7.930) | -4.601 (6.935) | -4.537 (7.322) | -5.506 (9.647) |
| **Legal advice services (Reference: No)** | | | | | | |
| **Yes** | 7.339 (12.035) | 7.351 (11.722) | 7.567 (6.832) | 7.893 (5.976) | 7.912 (6.309) | 7.621 (7.510) |
| **Urban** |  |  |  |  |  |  |
| **Daily living assistance (Reference: No)** | | |  |  |  |  |
| **Yes** | -0.409 (0.475) | -0.408 (0.455) | -0.406 (0.253) | -0.402 (0.186) ** | -0.402 (0.197) ** | -0.405 (0.203) ** |
| **Community-based health care (Reference: No)** | | |  |  |  |  |
| **Yes** | -0.037 (0.245) | -0.036 (0.237) | -0.022 (0.184) | 0.001 (0.198) | 0.003 (0.206) | -0.013 (0.121) |
| **Mental health support (Reference: No)** | | | | | | |
| **Yes** | -3.103 (11.928) | -3.142 (11.333) | -3.479 (6.708) | -3.977 (6.118) | -4.032 (6.720) | -3.557 (7.073) |
| **Legal advice services (Reference: No)** | | | | | | |
| **Yes** | -6.179 (13.992) | -6.012 (13.294) | -4.578 (7.874) | -2.464 (7.177) | -2.227 (7.883) | -4.251 (7.213) |
|  | **Total direct costs associated with informal care** | | | | |  |
| **Variables** | **0.1** | **0.3** | **0.5** | **0.7** | **0.9** | **Total rural/urban sample** |
| **Rural** |  |  |  |  |  |  |
| **Daily living assistance (Reference: No)** | | |  |  |  |  |
| **Yes** | -0.073 (0.809) | -0.069 (0.780) | 0.004 (0.316) | 0.055 (0.369) | 0.058 (0.387) | -0.007 (0.441) |
| **Community-based health care (Reference: No)** | | |  |  |  |  |
| **Yes** | 0.057 (0.448) | 0.056 (0.432) | 0.031 (0.175) | 0.014 (0.204) | 0.013 (0.214) | 0.035 (0.291) |
| **Mental health support (Reference: No)** | | | | | | |
| **Yes** | -0.675 (0.986) | -0.675 (0.950) | -0.684 (0.389) | -0.685 (0.411) | -0.685 (0.420) | -0.680 (0.401) |
| **Legal advice services (Reference: No)** | | | | | | |
| **Yes** | 0.049 (0.715) | 0.049 (0.689) | 0.068 (0.249) | 0.081 (0.289) | 0.081 (0.304) | 0.065 (0.302) |
| **Urban** |  |  |  |  |  |  |
| **Daily living assistance (Reference: No)** | | |  |  |  |  |
| **Yes** | 0.539 (1.106) | 0.527 (1.086) | 0.393 (0.984) | 0.274 (1.124) | 0.261 (1.151) | 0.401 (0.449) |
| **Community-based health care (Reference: No)** | | |  |  |  |  |
| **Yes** | -0.772 (1.221) | -0.772 (1.162) | -0.773 (0.461) * | -0.773 (0.372) ** | -0.773 (0.441) * | -0.773 (0.262) *** |
| **Mental health support (Reference: No)** | | | | | | |
| **Yes** | -0.143 (0.602) | -0.143 (0.574) | -0.152 (0.334) | -0.161 (0.349) | -0.162 (0.366) | -0.152 (0.318) |
| **Legal advice services (Reference: No)** | | | | | | |
| **Yes** | 0.016 (0.581) | 0.016 (0.554) | 0.014 (0.323) | 0.012 (0.336) | 0.012 (0.354) | 0.014 (0.340) |

Notes: Total rural sample is 6,307. Total urban sample is 6,207. Total direct costs include expenses related to transportation, medical supplies, and additional household needs. All the models control for needs-related variables and socioeconomic-related variables. Cells represent ﻿coefficient (robust standard errors). *** p<0.01, ** p<0.05, * p<0.1.
